# Effect of dose and duration of reduction in dietary sodium on blood pressure levels: systematic review and meta-analysis of randomised trials

**DOI:** 10.1136/bmj.m315

**Published:** 2020-02-24

**Authors:** Liping Huang, Kathy Trieu, Sohei Yoshimura, Bruce Neal, Mark Woodward, Norm R C Campbell, Qiang Li, Daniel T Lackland, Alexander A Leung, Cheryl A M Anderson, Graham A MacGregor, Feng J He

**Affiliations:** 1 Sydney School of Public Health, University of Sydney, Sydney, NSW, Australia; 2 The George Institute for Global Health, UNSW Sydney, Sydney, NSW, Australia; 3 National Cerebral and Cardiovascular Centre, Osaka, Japan; 4 Department of Epidemiology and Biostatistics, Imperial College London, London, UK; 5 The George Institute for Global Health, University of Oxford, Oxford, UK; 6 Departments of Medicine and Community Health Science, University of Calgary, Calgary, AB, Canada; 7 Medical University of South Carolina, Charleston, SC, USA; 8 The University of California, San Diego, CA, USA; 9 Wolfson Institute of Preventive Medicine, Barts and the London School of Medicine and Dentistry, Queen Mary University of London, London E1 4NS, UK

## Abstract

**Objective:**

To examine the dose-response relation between reduction in dietary sodium and blood pressure change and to explore the impact of intervention duration.

**Design:**

Systematic review and meta-analysis following PRISMA guidelines.

**Data sources:**

Ovid MEDLINE(R), EMBASE, and Cochrane Central Register of Controlled Trials (Wiley) and reference lists of relevant articles up to 21 January 2019.

**Inclusion criteria:**

Randomised trials comparing different levels of sodium intake undertaken among adult populations with estimates of intake made using 24 hour urinary sodium excretion.

**Data extraction and analysis:**

Two of three reviewers screened the records independently for eligibility. One reviewer extracted all data and the other two reviewed the data for accuracy. Reviewers performed random effects meta-analyses, subgroup analyses, and meta-regression.

**Results:**

133 studies with 12 197 participants were included. The mean reductions (reduced sodium *v* usual sodium) of 24 hour urinary sodium, systolic blood pressure (SBP), and diastolic blood pressure (DBP) were 130 mmol (95% confidence interval 115 to 145, P<0.001), 4.26 mm Hg (3.62 to 4.89, P<0.001), and 2.07 mm Hg (1.67 to 2.48, P<0.001), respectively. Each 50 mmol reduction in 24 hour sodium excretion was associated with a 1.10 mm Hg (0.66 to 1.54; P<0.001) reduction in SBP and a 0.33 mm Hg (0.04 to 0.63; P=0.03) reduction in DBP. Reductions in blood pressure were observed in diverse population subsets examined, including hypertensive and non-hypertensive individuals. For the same reduction in 24 hour urinary sodium there was greater SBP reduction in older people, non-white populations, and those with higher baseline SBP levels. In trials of less than 15 days’ duration, each 50 mmol reduction in 24 hour urinary sodium excretion was associated with a 1.05 mm Hg (0.40 to 1.70; P=0.002) SBP fall, less than half the effect observed in studies of longer duration (2.13 mm Hg; 0.85 to 3.40; P=0.002). Otherwise, there was no association between trial duration and SBP reduction.

**Conclusions:**

The magnitude of blood pressure lowering achieved with sodium reduction showed a dose-response relation and was greater for older populations, non-white populations, and those with higher blood pressure. Short term studies underestimate the effect of sodium reduction on blood pressure.

**Systematic review registration:**

PROSPERO CRD42019140812.

**Figure:**
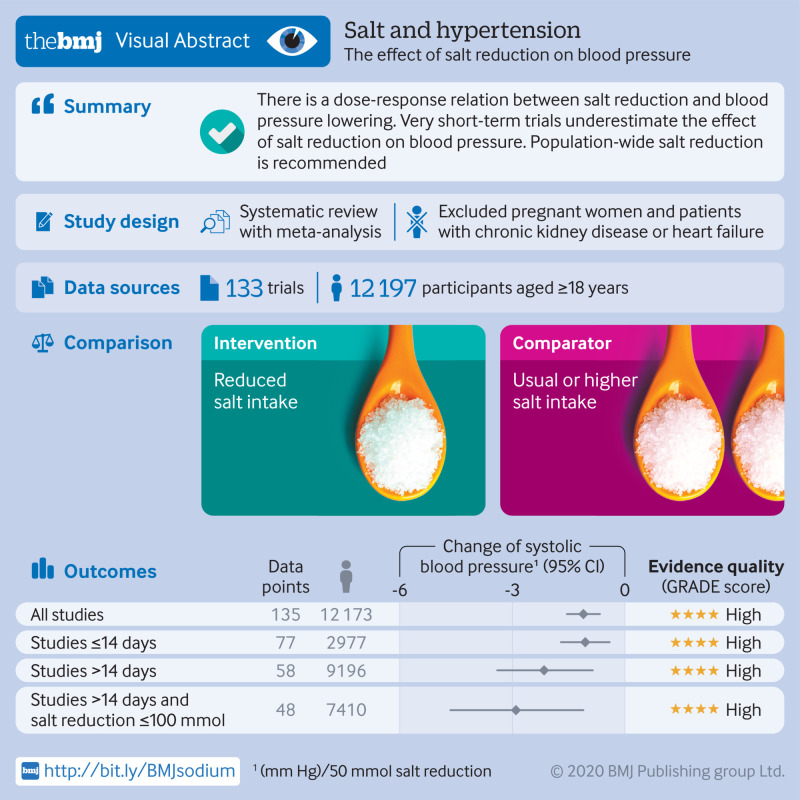


## Introduction

High blood pressure is a leading modifiable risk factor for cardiovascular disease, which caused at least 17.8 million deaths worldwide in 2017.^1^ A higher intake of dietary sodium is associated with a higher level of blood pressure in animals and humans.^2^ ^3^ ^4^ The physiological requirement for sodium in humans is less than 1 g a day,^5^ but currently most populations consume a much higher level.^6^ The maximum daily intake of dietary sodium recommended by the World Health Organisation (WHO) is 2 g (5 g salt) for adults,^7^ and most countries recommend reducing intake to less than 2.4 g a day^8^ ^9^ as part of a dietary approach to prevent high blood pressure and cardiovascular disease.

The effect of sodium reduction on blood pressure and the risk of cardiovascular disease has been examined in numerous studies. Although there is a consensus among health and scientific organisations to reduce intake of dietary sodium in the general population,^8^ ^9^ ^10^ a few scientists have claimed that the benefit of sodium restriction for populations with normal blood pressure is small^11^ ^12^ and could increase blood lipid levels and the risk of mortality.^12^ ^13^ ^14^ Others suggest that a higher risk of mortality at low sodium intake levels is an artefact attributable to factors such as reverse causation and biased estimation of sodium intake.^15^ ^16^

The nature of the association between change in sodium intake and blood pressure is key to understanding the potential for health interventions based on sodium reduction. Previous overviews of the data were limited because a definitive dose-response relation could not be determined, especially for participants with normal blood pressure.^12^ ^17^ ^18^ ^19^ A specific issue in previous meta-analyses was the inclusion of studies with sodium intake estimated from fractional urine samples.^11^ ^12^ ^19^ Fractional urine samples can produce overestimates of sodium intake when true intake is low but underestimates when true intake is high.^20^ Studies of short duration might also confound estimates of the average effect of change in sodium intake on blood pressure because large, short term reductions in sodium could elicit a different type of blood pressure response.^21^ A previous analysis that included 15 studies with measurements made at multiple time points was unable to determine whether effects of sodium reduction on blood pressure were sustained, declined, or increased with greater duration of intervention.^22^ The objective of this systematic review and meta-analysis was to examine the dose-response relation between dietary sodium reduction and blood pressure change, and to explore the impact of intervention duration, by applying more restricted inclusion criteria compared with previous reviews. The review was conducted with the support of the TRUE consortium.^23^

## Methods

### Search strategy

We carried out a search following a strategy developed for a previous meta-analysis^21^ that used keyword searches based on “sodium chloride, dietary,” “sodium, dietary,” or “diet, sodium-restricted” and “randomized controlled trial,” “controlled clinical trial,” or “randomized” (supplementary file 1). The databases searched included Ovid MEDLINE(R), EMBASE, and Cochrane Central Register of Controlled Trials (Wiley). The search date was from the start date of the databases to 21 January 2019. Additionally, we reviewed the references of pertinent original studies and review articles to identify additional studies. We imposed no language restriction on our search.

### Inclusion and exclusion criteria

Two of three reviewers (LH, KT, and SY) independently assessed records identified from the search for eligibility. We resolved any discrepancies by consensus. We included only trials with random allocation of participants to reduced dietary sodium intake and usual/higher dietary sodium intake (that is, control). Trials with concomitant interventions (eg, non-pharmacological interventions, antihypertensive or other drugs) were included only if the other interventions were applied equally to all randomised groups of interest. We included only studies with sodium intake estimated by 24 hour urine collection that also had data on systolic blood pressure or diastolic blood pressure measurements. Studies with only mean arterial blood pressure reported were not included unless we could retrieve relevant data from the authors. We excluded studies conducted in children (age <18 years), pregnant women, or individuals with confounding chronic conditions such as chronic kidney disease or heart failure.

### Study quality

Study quality was assessed independently by two reviewers based on the five domains defined by the Cochrane Collaboration’s tool for assessing risk of bias version 5.0.1^24^; namely, random sequence generation, allocation concealment, blinding of participants, personnel and outcome assessors, incomplete outcome data, and selective outcome reporting.

### Data extraction

One author (LH) extracted all data and two authors (KT and SY) reviewed the data for accuracy. Data sought for extraction included: characteristics of the study; demographics of the participants (race, mean age, percentage of female sex, percentage hypertension); study design (parallel group or crossover trial); risk of bias; duration of the intervention (calculated as the period from randomisation to the last follow-up measurement in parallel group studies and as the duration of each period of intervention, excluding run-in and washout, in crossover studies); 24 hour urinary sodium and blood pressure at baseline; and intervention effect on 24 hour urinary sodium and blood pressure.

For studies that only reported results as subgroups (eg, male and female subgroups), we obtained overall estimates as weighted averages based on the size of the subgroups for the primary analysis, following the Cochrane Handbook for Systematic Reviews of Interventions.^24^

Where available, data were extracted for subgroups defined by age, sex, ethnic group, and presence or absence of hypertension. We used casual blood pressure measurements rather than 24 hour ambulatory blood pressure if both were reported, and supine blood pressure was used ahead of standing blood pressure. In crossover studies, the last measurement of 24 hour urinary sodium and blood pressure at the highest sodium intake period was taken as the baseline.

The intervention effects on the 24 hour urinary sodium, systolic blood pressure, and diastolic blood pressure, were extracted directly from the studies, if reported. If not, we calculated them in crossover studies as the differences between the end of lowest sodium intake period (intervention) and the end of the highest sodium intake (control) period. For parallel studies, we calculated these as the differences between groups in the change from baseline to the last follow-up measurement.

Corresponding standard errors of each outcome were either extracted directly or calculated from standard deviations, confidence intervals, or exact P values following the Cochrane Handbook for Systematic Reviews of Interventions.^24^ If we could not derive the exact variance of the paired difference, we imputed it by assuming a correlation coefficient of 0.5 between the initial and final measurement following the same method used in a previous meta-analysis.^21^ We applied sensitivity analyses using different levels of correlation coefficients from 0.1 to 0.9 with 0.1 increments to check sensitivity to the assumption of a 0.5 correlation.

### Statistical analysis

We used random effects meta-analysis, based on the DerSimonian and Laird method, to generate pooled estimates of the intervention effect on 24 hour urinary sodium excretion, systolic blood pressure, and diastolic blood pressure. We used the I^2^ statistic to examine the heterogeneity between trial results and used funnel plots and Egger’s regression test to detect potential publication bias.

To explore the dose-response relation, we summarised the changes in systolic and diastolic blood pressure by categorising trials into five equal groups (quintiles) based on the change in 24 hour urinary sodium excretion. We plotted studies with more than two levels of dietary sodium intake in a connected line graph.

We assessed the effect of intervention duration on changes in blood pressure by grouping trials into five categories (≤7 days; >7 days to ≤14 days; >14 days to ≤30 days; >30 days to ≤6 months; >6 months). In addition, for studies with multiple follow-up measurements, we plotted the effects on blood pressure standardised to 50 mmol reduction of 24 hour urinary sodium against the duration of intervention in a connected line graph.

Age, sex, ethnic group, baseline sodium intake, and baseline blood pressure have been previously identified as potential modifiers of the effect of sodium reduction on blood pressure.^21^ We therefore summarised the effects on blood pressure for subgroups of trials (or subgroups within trials) defined by: mean age bands (≤35; >35 to ≤45; >45 to ≤55; >55 to ≤65; >65 years; unknown); sex (male; female; mixed; unknown); ethnicity (white; black; Asian; mixed race; unknown); mean baseline 24 hour sodium excretion (lowest <109 mmol; middle ≥109 to ≤209 mmol; highest >209 mmol)^25^; blood pressure status (normotensive; hypertensive; mixed; unknown); and mean baseline systolic blood pressure groups (<120; ≥120 to <130; ≥130 to <140; ≥140 to <150; ≥150 to <160, ≥160 mm Hg; unknown). The effects were standardised to a 50 mmol difference in 24 hour urinary sodium with variance estimated using the Taylor expansion.

To further explore the effects of these variables on blood pressure response to sodium reduction, we conducted unadjusted meta-regression for change in 24 hour urinary sodium, duration of intervention, mean age, percentage of white ethnicity, percentage of female sex, mean baseline blood pressure, and mean baseline 24 hour sodium excretion. We also did multivariable meta-regression including all covariates except for baseline 24 hour sodium excretion, which showed strong collinearity with the change in 24 hour urinary sodium (r=0.83). The following steps were undertaken to minimise missing data in the meta-regression analyses: if the studies did not report the participants’ race, this was imputed based on the study country (66 data points); if the percentage of female participants was not reported, the mean percentage of female of all other studies was used (8 data points); if only the age range was reported, the mean age was estimated as the mean of the minimum and maximum (12 data points); if neither mean age nor age range were reported, the mean age of all other studies was used (3 data points); and if baseline blood pressure was not reported, the mean value of all other studies was used (2 data points).

Short term studies are usually done using very restricted diets and could result in sudden large reductions in sodium intake that do not reflect medium or long term effects of sodium reduction on blood pressure.^21^ ^26^ Accordingly, we conducted stratified analyses for studies with intervention duration of 14 days or less versus more than 14 days. For the same reason, we also did an analysis restricted to trials longer than 14 days with a 100 mmol or smaller reduction in 24 hour urinary sodium. All analyses were done using Stata (version 15.1, StataCorp, TX).

### Patient and public involvement

Patients and the public were not involved in the design and conduct of this review.

## Results

**Fig 1:**
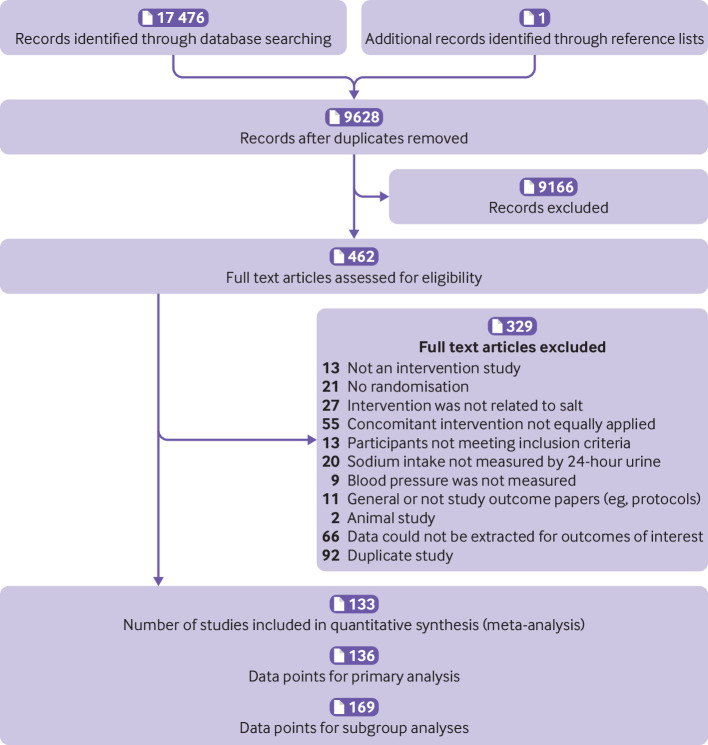
Flowchart for inclusion criteria

The search identified 17 477 records. After screening titles and abstracts, we selected 462 publications for full text review, of which 329 were excluded for the reasons summarised in figure 1. One hundred and thirty three studies^27^ ^28^ ^29^ ^30^ ^31^ ^32^ ^33^ ^34^ ^35^ ^36^ ^37^ ^38^ ^39^ ^40^ ^41^ ^42^ ^43^ ^44^ ^45^ ^46^ ^47^ ^48^ ^49^ ^50^ ^51^ ^52^ ^53^ ^54^ ^55^ ^56^ ^57^ ^58^ ^59^ ^60^ ^61^ ^62^ ^63^ ^64^ ^65^ ^66^ ^67^ ^68^ ^69^ ^70^ ^71^ ^72^ ^73^ ^74^ ^75^ ^76^ ^77^ ^78^ ^79^ ^80^ ^81^ ^82^ ^83^ ^84^ ^85^ ^86^ ^87^ ^88^ ^89^ ^90^ ^91^ ^92^ ^93^ ^94^ ^95^ ^96^ ^97^ ^98^ ^99^ ^100^ ^101^ ^102^ ^103^ ^104^ ^105^ ^106^ ^107^ ^108^ ^109^ ^110^ ^111^ ^112^ ^113^ ^114^ ^115^ ^116^ ^117^ ^118^ ^119^ ^120^ ^121^ ^122^ ^123^ ^124^ ^125^ ^126^ ^127^ ^128^ ^129^ ^130^ ^131^ ^132^ ^133^ ^134^ ^135^ ^136^ ^137^ ^138^ ^139^ ^140^ ^141^ ^142^ ^143^ ^144^ ^145^ ^146^ ^147^ ^148^ ^149^ ^150^ ^151^ ^152^ ^153^ ^154^ ^155^ ^156^ ^157^ ^158^ ^159^ met our inclusion criteria, with 136 data points extracted for primary analyses involving 12 197 participants (fig 1) and 169 data points for various subgroup analyses. The characteristics of the included studies are presented in supplementary file 2. Fifty seven per cent (77/136) data points had intervention durations of up to 14 days, 21% (28/136) had intervention durations between 15 days and 30 days, 19% (26/136) had intervention durations longer than 30 days but within six months, and 4% (5/136) had intervention durations longer than six months.

**Fig 2:**
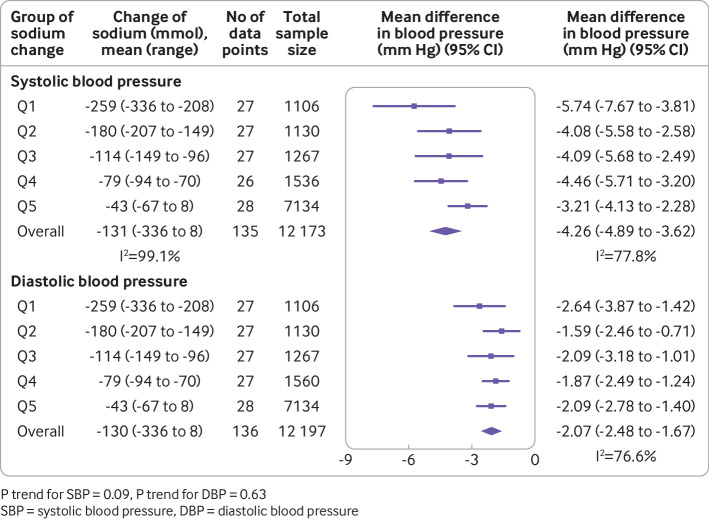
Association of magnitude of sodium reduction (mmol) with size of blood pressure reduction (mm Hg)

**Fig 3:**
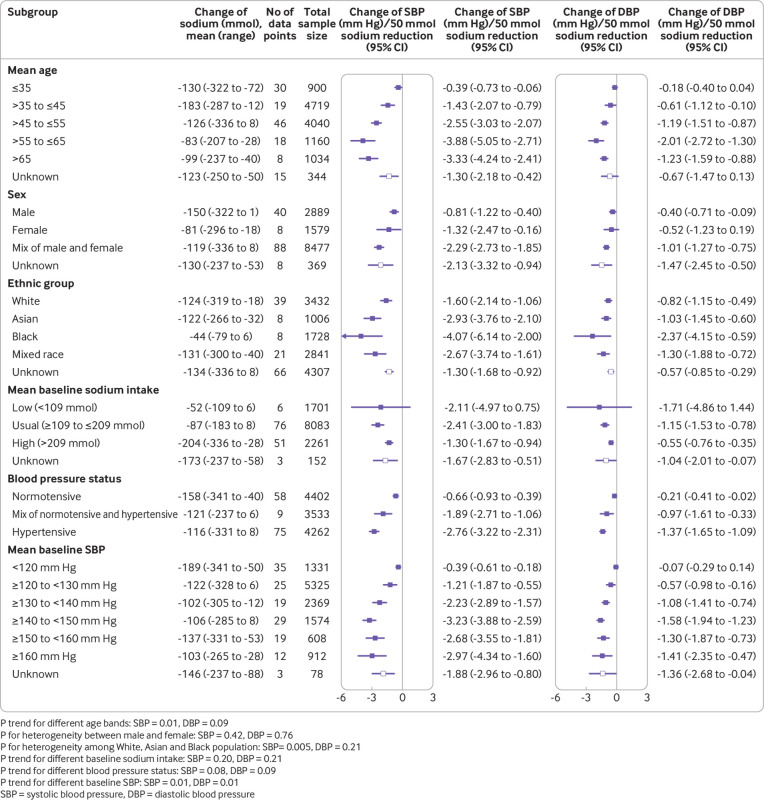
Effects of age, sex, ethnicity, baseline sodium intake, baseline blood pressure, and blood pressure status on the size of blood pressure reduction (mm Hg) achieved with a 50 mmol reduction in sodium excretion

Overall, we saw a mean change of −130 mmol (95% confidence interval −145 to −115, P<0.001, I^2^=99.1%) in 24 hour urinary sodium, −4.26 mm Hg (−4.89 to −3.62, P<0.001, I^2^=77.8%) in systolic blood pressure, and −2.07 mm Hg (−2.48 to −1.67, P<0.001, I^2^=76.6%) in diastolic blood pressure (fig 2). We used different correlation coefficients to calculate the variance, which yielded similar pooled estimates of difference in systolic blood pressure (ranging from −4.59 mm Hg (−5.27 to −3.91) to −4.18 mm Hg (−4.59 to −3.51)) and diastolic blood pressure (ranging from −2.04 mm Hg (−2.46 to −1.63) to −2.13 mm Hg (−2.58 to −1.67)). Sodium reduction was associated with separately statistically significant reductions in blood pressure for most subgroups studied. As reflected by the I^2^ values, the magnitude of effect varied substantially between contributing trials, as well as between many of the subgroups (fig 3).

### Association of magnitude of sodium reduction with size of blood pressure reduction

The meta-analysis of trials by group of achieved sodium reduction (fig 2) identified no clear association between the magnitude of sodium reduction and magnitude of either the systolic blood pressure reduction (P trend=0.09) or the diastolic blood pressure reduction (P trend=0.63). Likewise, the univariable meta-regression analyses including all studies (table 1) showed no association of blood pressure effect with magnitude of sodium reduction. However, the magnitude of the change in 24 hour urinary sodium excretion was positively associated with the change in blood pressure after adjusting for intervention duration, mean age, percentage of female sex, percentage of white ethnicity, and baseline blood pressure in the multivariable meta-regression. In these analyses, each 50 mmol reduction in 24 hour urinary sodium was associated with a 1.10 mm Hg (0.66 to 1.54) decrease in systolic blood pressure and a 0.33 mm Hg (0.04 to 0.63) decrease in diastolic blood pressure. Sensitivity analyses excluding studies with missing data other than ethnicity (18 studies) did not change the results substantially. We did not exclude studies missing only ethnicity data because this could be estimated with high confidence based on the study country. The analysis of studies with more than two levels of dietary sodium intake provided further support for a dose-response association between magnitude of sodium reduction and magnitude of systolic blood pressure response (supplementary fig 1). Findings in the subsidiary analyses for DBP were less clear.

**Table 1:** Coefficient statistics of unadjusted and multivariable meta-regression on the association between blood pressure change and 24 hour urinary sodium change and other covariates

Dependent variables and studies	Independent variables	Unadjusted		Multivariable	
		Coefficient* (95% CI)	P value	Coefficient* (95% CI)	P value
Change in SBP (mm Hg)					
All studies (135 data points)	Change of 24 hour urinary sodium (mmol)	0.007 (−0.002 to 0.016)	0.106	0.022 (0.013 to 0.031)	<0.001
	Duration of intervention (days)	−0.003 (−0.006 to 0.001)	0.124	0.000 (−0.003 to 0.003)	0.849
	Mean age (years)	0.121 (0.073 to 0.169)	<0.001	0.111 (0.046 to 0.177)	0.001
	Percentage of female sex	2.258 (−0.794 to 5.309)	0.146	−0.412 (−3.170 to 2.346)	0.768
	Percentage of white ethnicity	−3.039 (−5.397 to −0.680)	0.012	−2.957 (−5.000 to −0.913)	0.005
	Mean baseline SBP (mm Hg)	0.102 (0.065 to 0.138)	<0.001	0.074 (0.030 to 0.118)	0.001
Studies with ≤14 days' intervention (77 data points)	Change of 24 hour urinary sodium (mmol)	0.015 (−0.001 to 0.030)	0.064	0.021 (0.008 to 0.034)	0.002
	Duration of intervention (days)	0.208 (−0.084 to 0.500)	0.160	0.071 (−0.182 to 0.324)	0.577
	Mean age (years)	0.180 (0.106 to 0.254)	<0.001	0.105 (−0.006 to 0.217)	0.064
	Percentage of female sex	2.701 (−1.438 to 6.841)	0.198	−0.233 (−4.031 to 3.566)	0.903
	Percentage of white ethnicity	−5.061 (−8.923 to −1.199)	0.011	−2.787 (−6.343 to 0.770)	0.123
	Mean baseline SBP (mm Hg)	0.132 (0.080 to 0.185)	<0.001	0.076 (0.002 to 0.150)	0.044
Studies with >14 days' intervention (58 data points)	Change of 24 hour urinary sodium (mmol)	0.043 (0.019 to 0.067)	0.001	0.043 (0.017 to 0.068)	0.002
	Duration of intervention (days)	−0.003 (−0.006 to −0.001)	0.016	0.000 (−0.002 to 0.003)	0.813
	Mean age (years)	0.107 (0.013 to 0.201)	0.027	0.107 (0.012 to 0.203)	0.028
	Percentage of female sex	0.523 (−4.924 to 5.970)	0.848	−0.421 (−5.337 to 4.496)	0.864
	Percentage of white ethnicity	−1.251 (−3.973 to 1.471)	0.361	−3.342 (−6.071 to −0.614)	0.017
	Mean baseline SBP (mm Hg)	0.067 (0.004 to 0.129)	0.036	0.058 (−0.003 to 0.120)	0.062
Studies with duration >14 days and sodium reduction <=100 mmol (48 data points)	Change of 24 hour urinary sodium (mmol)	0.059 (0.023 to 0.094)	0.002	0.058 (0.022 to 0.093)	0.002
	Duration of intervention (days)	−0.003 (−0.005 to −0.001)	0.007	0.001 (−0.001 to 0.003)	0.410
	Mean age (years)	0.113 (0.029 to 0.198)	0.010	0.110 (0.032 to 0.188)	0.007
	Percentage of female sex	0.945 (−4.278 to 6.169)	0.717	0.155 (−3.854 to 4.164)	0.938
	Percentage of white ethnicity	−1.302 (−3.943 to 1.340)	0.326	−3.497 (−5.724 to −1.271)	0.003
	Mean baseline SBP (mm Hg)	0.083 (0.025 to 0.141)	0.006	0.087 (0.035 to 0.140)	0.002
Change in DBP (mm Hg)					
All studies (136 data points)	Change of 24 hour urinary sodium (mmol)	0.000 (−0.006 to 0.006)	0.972	0.007 (0.001 to 0.013)	0.028
	Duration of intervention (days)	−0.001 (−0.003 to 0.001)	0.555	0.000 (−0.002 to 0.002)	0.980
	Mean age (years)	0.068 (0.036 to 0.100)	<0.001	0.041 (−0.002 to 0.084)	0.062
	Percentage of female sex	−0.373 (−2.327 to 1.582)	0.707	−1.480 (−3.343 to 0.383)	0.118
	Percentage of white ethnicity	−1.102 (−2.569 to 0.366)	0.140	−0.909 (−2.249 to 0.430)	0.182
	Mean baseline DBP (mm Hg)	0.102 (0.071 to 0.133)	<0.001	0.088 (0.049 to 0.127)	<0.001
Studies with ≤14 days' intervention (77 data points)	Change of 24 hour urinary sodium (mmol)	0.003 (−0.007 to 0.014)	0.531	0.007 (−0.002 to 0.015)	0.134
	Duration of intervention (days)	0.302 (0.127 to 0.478)	0.001	0.174 (0.006 to 0.343)	0.043
	Mean age (years)	0.104 (0.055 to 0.154)	<0.001	0.021 (−0.054 to 0.095)	0.586
	Percentage of female sex	−0.381 (−3.011 to 2.249)	0.774	−1.357 (−3.722 to 1.007)	0.256
	Percentage of white ethnicity	−1.230 (−3.590 to 1.130)	0.302	−0.570 (−2.701 to 1.561)	0.596
	Mean baseline DBP (mm Hg)	0.126 (0.086 to 0.166)	<0.001	0.099 (0.036 to 0.162)	0.003
Studies with >14 days' intervention (59 data points)	Change of 24 hour urinary sodium (mmol)	0.015 (0.001 to 0.029)	0.035	0.013 (−0.002 to 0.029)	0.094
	Duration of intervention (days)	−0.001 (−0.003 to 0.000)	0.157	0.000 (−0.002 to 0.002)	0.983
	Mean age (years)	0.022 (−0.039 to 0.083)	0.472	0.035 (−0.033 to 0.103)	0.308
	Percentage of female sex	−1.213 (−4.464 to 2.039)	0.458	−1.263 (−4.617 to 2.091)	0.453
	Percentage of white ethnicity	−0.780 (−2.472 to 0.911)	0.359	−1.493 (−3.353 to 0.368)	0.113
	Mean baseline DBP (mm Hg)	0.049 (−0.011 to 0.109)	0.110	0.048 (−0.015 to 0.110)	0.134
Studies with duration >14 days and sodium reduction ≤100 mmol (49 data points)	Change of 24 hour urinary sodium (mmol)	0.018 (−0.002 to 0.037)	0.081	0.013 (−0.010 to 0.035)	0.258
	Duration of intervention (days)	−0.001 (−0.002 to 0.000)	0.049	0.000 (−0.002 to 0.001)	0.810
	Mean age (years)	0.032 (−0.019 to 0.082)	0.210	0.041 (−0.017 to 0.099)	0.162
	Percentage of female sex	−1.343 (−4.122 to 1.437)	0.336	−0.753 (−3.557 to 2.050)	0.591
	Percentage of white ethnicity	−0.370 (−1.848 to 1.109)	0.617	−0.979 (−2.544 to 0.586)	0.214
	Mean baseline DBP (mm Hg)	0.059 (0.006 to 0.111)	0.029	0.058 (0.003 to 0.113)	0.040

SBP=systolic blood pressure; DBP=diastolic blood pressure.

* The coefficient means the change of the dependent variable (change in SBP or DBP) with each unit increase of the independent variables.

### Association of duration of sodium reduction intervention with size of blood pressure reduction

**Fig 4:**
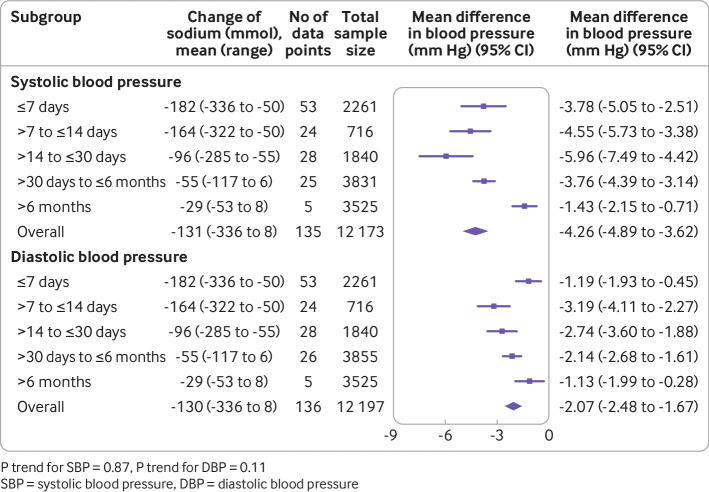
Association of duration of sodium reduction intervention with size of blood pressure reduction (mm Hg)

The meta-analysis of trials by intervention duration (fig 4) identified no overall association between the duration of the sodium reduction intervention and the magnitude of either the systolic blood pressure reduction (P trend=0.87) or the diastolic blood pressure reduction (P trend=0.11). Likewise, the univariable meta-regression analyses of all trials showed no association of blood pressure effect with duration of sodium reduction and neither did the multivariable meta-regressions. The six studies that recorded multiple measurements at different time points showed no apparent difference in the pattern of blood pressure lowering over time (supplementary fig 2), which corresponded to the meta-regression analyses. In the subsidiary analysis of studies with intervention duration >14 days compared with ≤14 days, the effect of each 50 mmol reduction in 24 hour urinary sodium excretion on systolic blood pressure reduction was approximately twice as large in the studies of longer intervention duration (2.13 mm Hg; 0.85 to 3.40 *v* 1.05 mm Hg; 0.40 to 1.70). The univariable meta-regression analyses restricted to longer trials and trials with smaller reductions in sodium excretion showed inverse associations between intervention duration and magnitude of systolic blood pressure reduction but these were not apparent in the corresponding multivariable meta-regression analyses (table 1) with I^2^ reduced to 24.9%. There was no consistent pattern of association between intervention duration and reduction in diastolic blood pressure.

### Association of age, sex, race, baseline sodium intake, and baseline blood pressure with the size of blood pressure fall with 50 mmol sodium reduction

The analysis of trial subgroups standardised to a 50 mmol sodium reduction (fig 3) identified positive associations between the magnitude of systolic blood pressure reduction and baseline age and baseline systolic blood pressure (both P trend=0.01), and statistically significant heterogeneity by race (P=0.005). We observed no association for baseline sodium intake (P trend=0.20) or blood pressure status (P trend=0.08) and there was no detectable heterogeneity by sex (P=0.42). The meta-regression analyses (table 1) provided support for independent effects of age, ethnicity, and baseline blood pressure as modifiers of the effect of sodium reduction on blood pressure.

### Study quality and publication bias

Most studies did not report whether there was random sequence generation (80.1%, 109/136) or appropriate allocation concealment (83.8%, 114/136). Only 41.9% (57/136) studies were double blinded and 52.9% (72/136) had inadequate blinding (open studies, or with only participants or outcome observers blinded). Most studies had low rates of loss to follow-up and there was low risk of selective outcome reporting (supplementary figure 3, supplementary file 3). Although the information for assessing individual study quality was limited, the overall evidence should be considered of fairly high quality since only randomised trials were included and only a small proportion of studies had significant missing data.

Egger’s regression test suggested asymmetry of funnel plots for both systolic blood pressure change (P<0.001) and diastolic blood pressure change (P=0.005) (supplementary figure 4). Use of the trim and fill method did not change the results and the contour enhanced funnel plots did not suggest under-reporting of studies with less statistical significance (supplementary figure 5). Asymmetry of the funnel plots is more likely to arise from other differences in study characteristics.

## Discussion

### Principal findings

This meta-analysis shows that sodium reduction leads to a significant reduction in systolic blood pressure in adults, both female and male, all ethnic groups, and in both hypertensive and normotensive populations. Diastolic blood pressure also decreased significantly in most participant subgroups. There was a dose-response relation with a greater reduction in sodium intake producing a greater fall in blood pressure. Populations with older age and higher baseline blood pressure achieved greater blood pressure lowering from the same amount of sodium reduction and so did non-white compared with white populations.

Overall, the duration of the sodium reduction intervention was not associated with the size of the change in blood pressure, although short term studies of less than 15 days’ duration appear to underestimate the effect of sodium reduction on blood pressure. With few long term studies available, additional research is required to draw a definitive conclusion about whether prolonged sodium reduction influences the magnitude of the blood pressure lowering.

### Strengths and limitations of the study

This meta-analysis represents a substantial update and enhancement compared with previous overviews. The selective inclusion of studies that used 24 hour urine collections to estimate intervention effect on sodium intake reduced the risk of bias, while the inclusion of studies with concomitant interventions applied in the same way to both intervention and control arms maximised the available data and the statistical power of the analyses. Likewise, the inclusion of studies regardless of the length of the duration of intervention enabled a robust and powerful examination of the effects of intervention duration on outcomes. The extensive sensitivity analyses provided for a full understanding of the strengths and weakness of the findings. There were, however, limitations in regard to our capacity to assess the quality of the studies. There was substantial heterogeneity across the included studies, but this was largely explained by some the of study characteristics in the meta-regression analyses. When the analyses were limited to studies with duration of more than 14 days and a sodium reduction of up to 100 mmol, the I^2^ statistics for heterogeneity reduced to 24.9%. The use of study level data rather than individual participant data greatly reduced the power of the analyses, although this limitation was somewhat offset by the large number of studies available. Different studies defined hypertension using different criteria and there was limited capacity to quantify the effects of pharmaceutical treatments on baseline diagnoses of hypertension or baseline blood pressure measurements. Finally, our use of random effects meta-analysis has resulted in wider confidence intervals but might better reflect uncertainty about the true constancy of effects across included trials.

### Comparison with other studies

#### Effect of sodium reduction on blood pressure and the dose-response relation

The overall effect of sodium reduction on blood pressure has been observed in several previous meta-analyses, despite different trial selection criteria.^12^ ^21^ ^160^ ^161^ We also observed strong associations of the magnitude of sodium reduction with the magnitude of the fall in systolic blood pressure,^11^ ^21^ and interactions of age, race, and baseline blood pressure with size of the systolic blood pressure fall, as shown in previous reviews.^162^ ^163^ However, the 2.2 mm Hg reduction in systolic blood pressure for each 100 mmol reduction in 24 hour urinary sodium observed in the current overview is substantially less compared with the 3.83 mm Hg reduction reported in a previous overview.^164^ The difference may be owing to the inclusion of studies with sodium intake estimated from fractional urine collections in the previous review.^164^ Another overview that included only studies with at least four weeks’ intervention and only moderate 24 hour urinary sodium reduction identified a 5.8 mm Hg reduction in SBP for each 100 mmol reduction in sodium,^21^ which is similar to the effect found in our subgroup analysis of studies longer than 14 days with a sodium reduction ≤100 mmol, further highlighting the sensitivity of the estimated strength of the dose-response association to the type of studies included in the analysis.

Previous overviews have generated uncertainty regarding the effects of sodium reduction among individuals with different levels of starting blood pressure. Some reports have suggested much larger effects in hypertensive individuals compared with non-hypertensive individuals,^21^ while others suggested that sodium reduction is of value only in those with hypertension.^11^ ^12^ The conclusion that there is no value in non-hypertensive individuals is dependent upon the results from very short term studies in which sodium reduction had a limited effect on blood pressure and in which there were adverse effects on other markers of cardiovascular risk. The responses of the renin-angiotensin system and sympathetic nervous system as well as adverse metabolic effects associated with acute large falls in dietary sodium do not, however, appear to be present in longer term interventions^21^ ^165^ and it is unlikely that short term unfavourable metabolic effects would override the long term benefits anticipated from sustained blood pressure lowering of moderate magnitude. Our review identifies an approximate doubling of the effect of sodium reduction on blood pressure in studies of longer than two weeks’ duration versus shorter studies, indicating that the full effects of dietary sodium reduction require several weeks to become apparent. Very short term studies of sodium reduction are not a sound basis for drawing conclusions about the effects of sodium reduction on blood pressure and are not helpful in formulating policy recommendations for public health.

Analyses that simply separate studies based upon those that included hypertensive, non-hypertensive, or mixed populations are weak because the definition of hypertension is arbitrary and there is a rationale for expecting a graded interaction between sodium reduction, blood pressure reduction, and starting blood pressure. In the present analysis, meta-regressions based on mean starting blood pressure levels of participants in each study provided for a much more nuanced evaluation of the effects of starting blood pressure on the size of the blood pressure fall achieved with sodium reduction. These analyses showed that sodium reduction produced a progressively greater reduction in blood pressure among those with higher starting blood pressure levels, but also that sodium reduction substantially lowered blood pressure, even among those with starting systolic blood pressure levels as low as 120 mm Hg. These findings indicate potentially important health benefits from sodium reduction among normotensive as well as hypertensive individuals. More importantly, sodium reduction among normotensive individuals could potentially avert or delay the development of hypertension with ageing as the association between sodium intake and blood pressure is greater at older age.^3^

The differential blood pressure lowering effect of sodium reduction across different ethnic groups has been observed in various studies and meta-analyses^12^ ^21^ ^166^; specifically, there was a greater blood pressure reduction in non-white populations compared with white populations for the same amount of sodium reduction. Some authors explained this phenomenon as caused by differential “salt sensitivity.”^167^ Others have shown that the difference in the responsiveness of the renin-angiotensin system to sodium reduction among various ethnic groups is at least partially responsible.^166^ Nonetheless, while population wide sodium reduction is recommended, the cost effectiveness for some particular populations is potentially greater. This has important public health implications, especially in regions where resources are constrained.

The findings from this overview of randomised trials conflict directly with findings from the Prospective Urban Rural Epidemiology (PURE)^168^ study, which reported that associations between sodium intake and systolic blood pressure are observed only among communities with very high sodium intake (>5.08 g or 221 mmol sodium/day, equivalent to 13 g/day salt). We observed very clear effects of sodium reduction on both systolic and diastolic blood pressure at levels of sodium intake far below this. Measurement errors and uncontrolled confounding in the PURE study have likely biased conclusions about the association of sodium intake and blood pressure.^16^ ^169^

#### Impact of intervention duration of sodium reduction

The optimal method to assess the impact of duration of sodium reduction on the magnitude of blood pressure reduction would be to collate data from studies that measure change of sodium and change of blood pressure at multiple time points. In practice, however, most studies made measurements only once at completion of follow-up. Neither the univariable nor the multivariable meta-regressions identified an effect of intervention duration on the size of the blood pressure fall achieved with sodium reduction. The power of the analyses was strengthened by the wide range of intervention durations recorded (from three days to five years) but limited by the highly skewed distribution of the studies, with most being of short or very short duration. Among the 133 studies included, 77 were shorter than 15 days’ duration and only five extended beyond six months. The DASH sodium trial that assessed effects at four time points over one month showed with some rigour that blood pressure effects from sodium reduction were clearly greater at week 4 compared with earlier weeks.^170^

## Conclusions and policy implications

Sodium reduction resulted in lower blood pressure among a very broad group of populations with a strong dose-response relation between the magnitude of the sodium reduction achieved and the magnitude of the fall in blood pressure. The effects of sodium reduction were more evident at higher starting blood pressure levels, older ages, and among non-white populations, but almost every population group examined achieved a reduction in blood pressure. In trials of more than two weeks’ duration, the dose-response relation between sodium reduction and blood pressure fall was greater than that in trials of shorter duration, but there was limited evidence that interventions of longer duration further increased the effects of sodium reduction on blood pressure. Longer term trials that achieve sustained sodium reduction and make multiple assessments of blood pressure are required to properly assess this issue.

**What is already known on this topic**

An extensive body of evidence has shown that a higher level of dietary sodium intake is associated with a higher blood pressure

There are clear effects of sodium reduction on blood pressure in those with hypertension, but uncertainty persists about the comparability of effects in different population subsets. In addition, the impact of intervention duration is not fully understood

**What this study adds**

Evidence shows that sodium reduction lowers blood pressure in both hypertensive and non-hypertensive individuals, with greater effects in high risk subsets

The magnitude of blood pressure lowering achieved with sodium reduction showed a dose-response relation

Very short term trials could substantially underestimate the effect of sodium reduction on blood pressure

## Notes

Contributors: Members of the TRUE Consortium conceived the review. LH undertook the search. LH, KT, and SY undertook the screening for eligibility in duplicate. LH extracted all relevant data from published papers. KT and SY checked all extracted data for accuracy. MW and QL provided advice on statistical analyses. LH analysed the data and drafted the manuscript. FH and BN provided overall guidance and editing of the manuscript. All authors in the author list provided comments and feedback on the manuscript, and GM and NC also provided some guidance during the conduct of the review. The corresponding author attests that all listed authors meet authorship criteria and that no others meeting the criteria have been omitted. LH and FH are the guarantors of this manuscript.

Ethical approval: Ethical approval was not required for this research.

Data sharing: Data used for analysis have been included in this manuscript. Additional data can be obtained upon request.

The lead author (the manuscript’s guarantor) affirms that the manuscript is an honest, accurate, and transparent account of the study being reported; that no important aspects of the study have been omitted; and that any discrepancies from the study as planned (and, if relevant, registered) have been explained.

Dissemination to participants and related patient and public communities: Following publication, the results of this review will be disseminated to appropriate audiences such as academia, clinicians, policy makers, and the general public, through various channels including engagement with collaborators and World Action on Salt and Health (WASH) members, press release, social media, e-newsletter, WHO Collaborating Centre on Salt Reduction website and monthly bulletin.

## Web extra

material supplied by authors

**Web appendix**: Supplementary figures 1-5

**Web appendix**: supplementary files 1-3
