# Supplementary material for: Effect of dose and duration of reduction in dietary sodium on blood pressure levels: systematic review and meta-analysis of randomised trials
Source: BMJ. 2020 Feb 24;368:m315. doi: 10.1136/bmj.m315 (PMC7190039; doi:10.1136/bmj.m315)

Supplementary figure 1 Mean systolic (right) and diastolic (left) blood pressure (mm Hg) at different levels of sodium intake in 7 studies

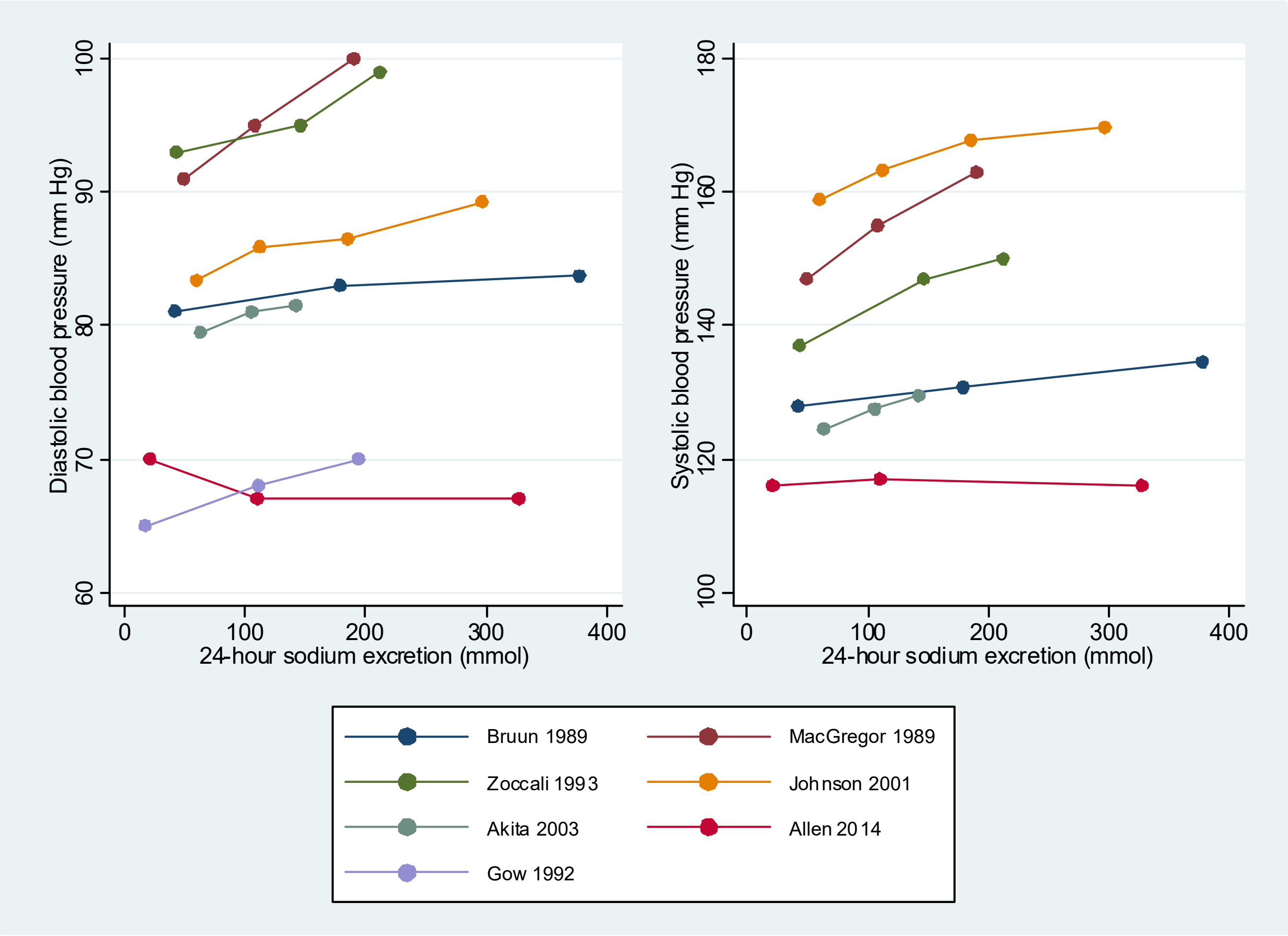

Supplementary figure 2 Change of systolic (right) and diastolic (left) blood pressure (mm Hg) for each 50 mmol change in sodium intake at different follow up times in 6 studies.

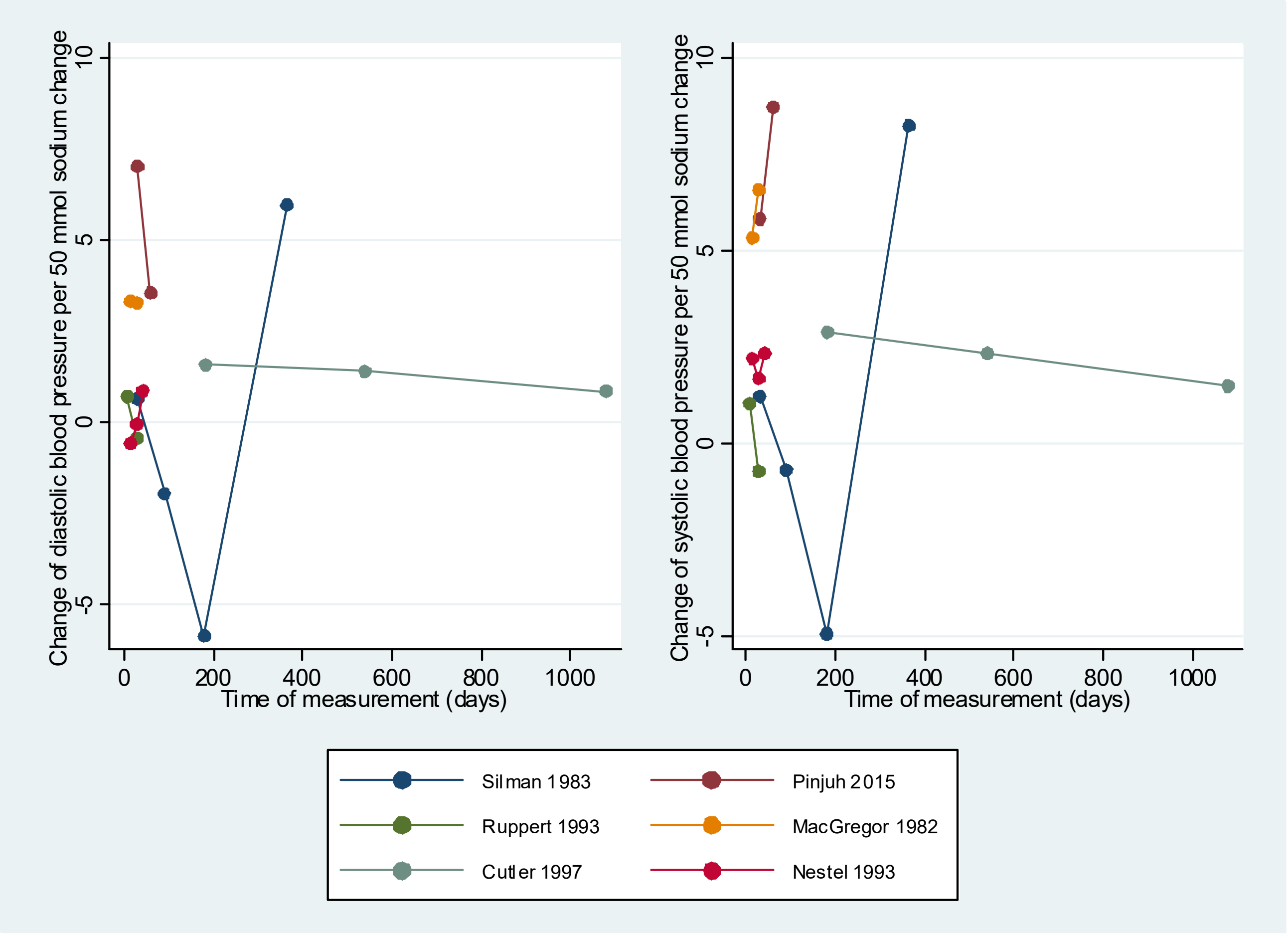

Supplementary figure 3 Study quality

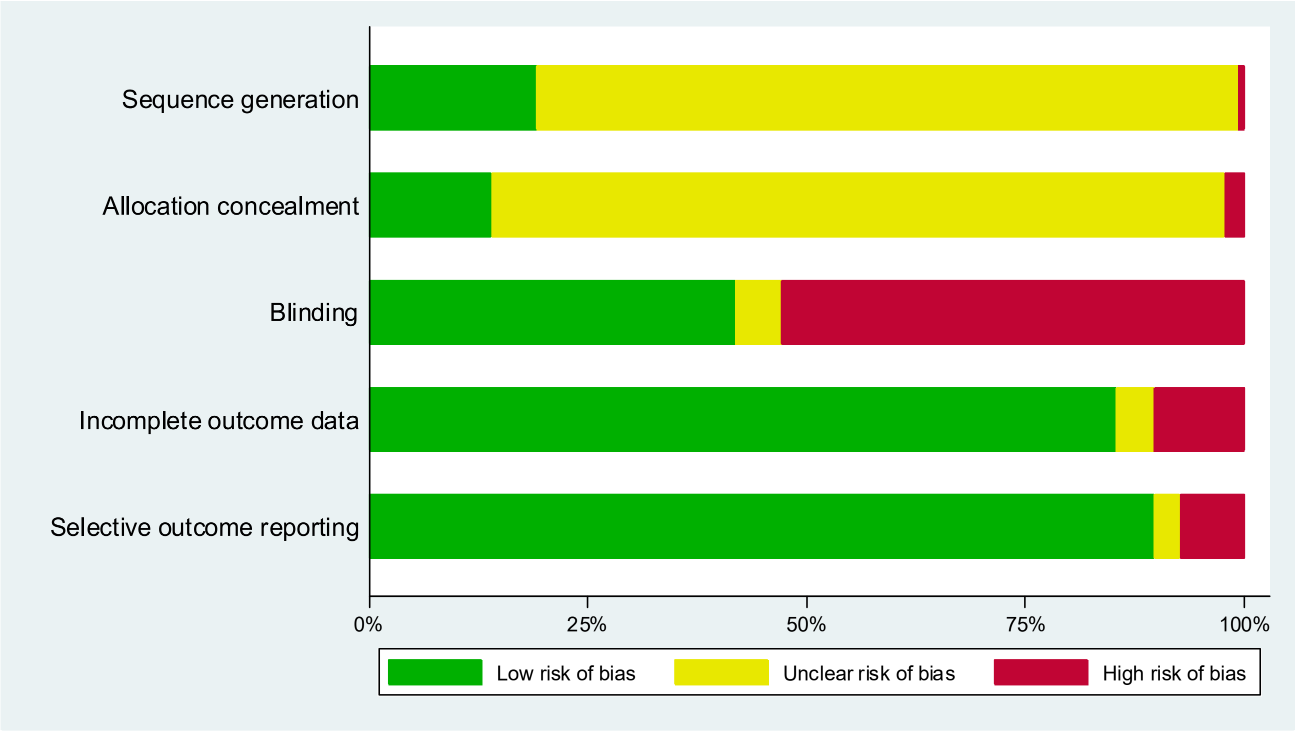

Supplementary figure 4 Funnel plot of systolic blood pressure (A) and diastolic blood pressure (B) change

A

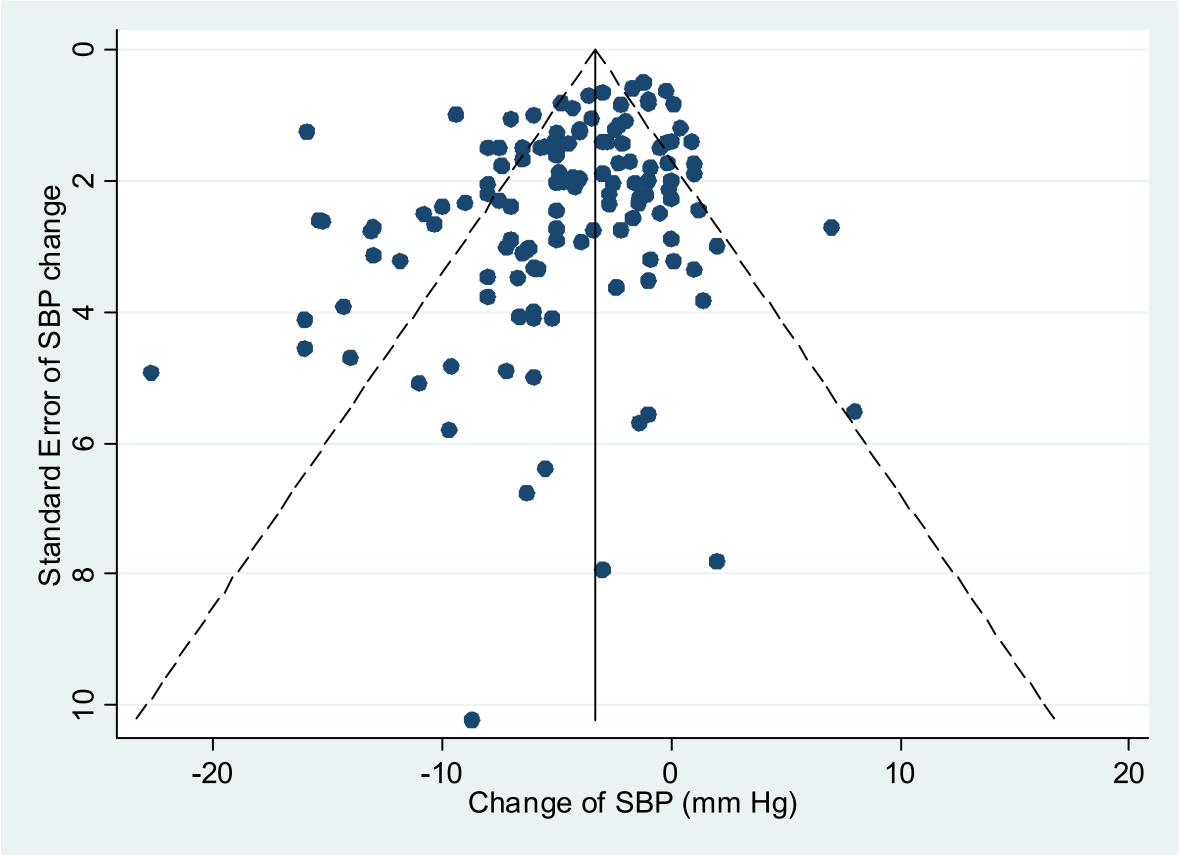

B

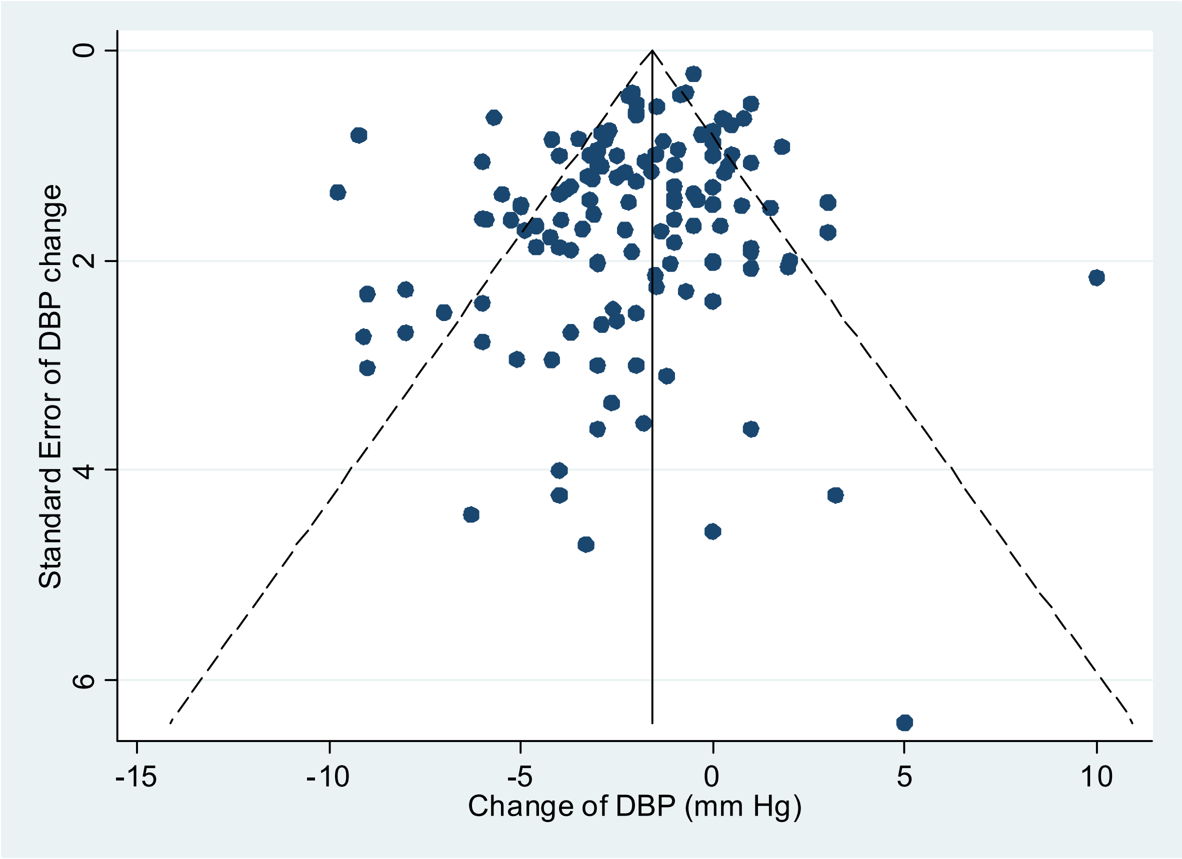

Supplementary figure 5 Contour-enhanced funnel plots for SBP change (A) and DBP change (B)

A

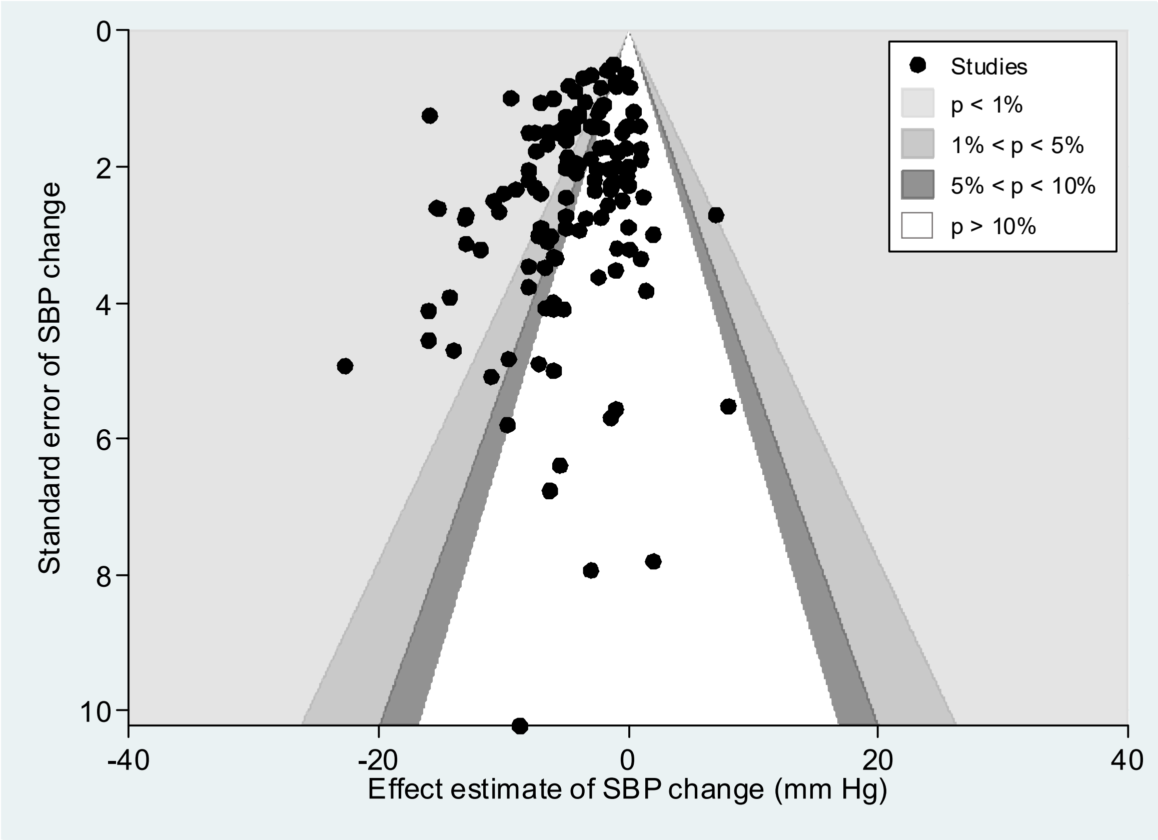

B

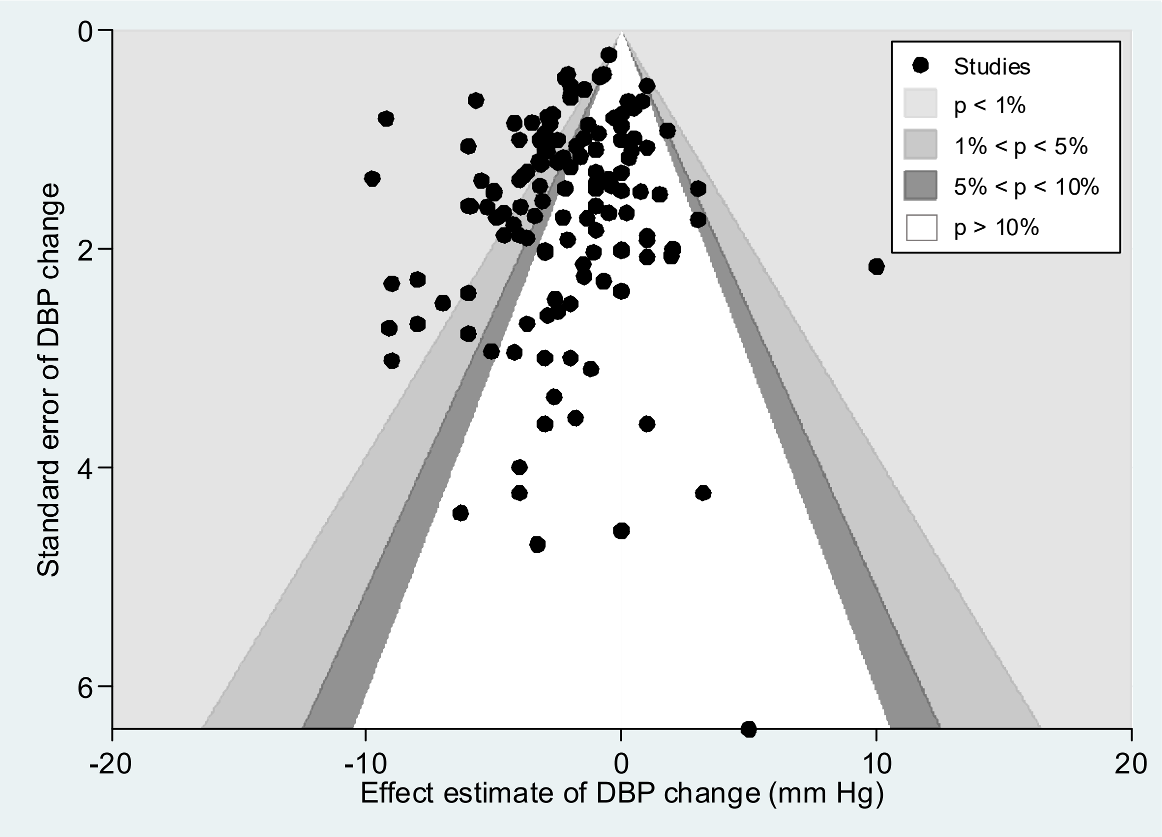

Supplement: Supplementary file 1 — Web appendix: Supplementary figures 1-5 [file hual051597.ww1.pdf]
