# Supplementary material for: Effect of dose and duration of reduction in dietary sodium on blood pressure levels: systematic review and meta-analysis of randomised trials
Source: BMJ. 2020 Feb 24;368:m315. doi: 10.1136/bmj.m315 (PMC7190039; doi:10.1136/bmj.m315)
Supplement: Supplementary file 2 — Web appendix: supplementary files 1-3 [file hual051597.ww2.pdf]

Supplementary file 1 Search Strategy to identify randomised salt reduction trials

Ovid MEDLINE(R) from 1946 to January 2019

----

1 sodium chloride, dietary/
2 exp sodium, dietary/
3 diet, sodium-restricted/
4 ((sodium or salt) adj3 (restrict$ or curb$ or limit$ or minim$ or low$ or reduc$ or intake or diet$ or free)).tw.
5 or/1-4
6 randomized controlled trial.pt.
7 controlled clinical trial.pt.
8 randomized.tw.
9 placebo.tw.
10 drug therapy/
11 randomly.tw.
12 trial.tw.
13 groups.tw.
14 or/6-13
15 animals/ not (humans/ and animals/)
16 14 not 15
17 5 and 16
18 remove duplicates from 17

EMBASE from 1946 to January 2019

----

1 sodium chloride, dietary/
2 sodium intake/
3 sodium restriction/
4 ((sodium or salt) adj3 (restrict$ or curb$ or limit$ or minimi$ or low$ or reduc$ or intake or diet$ or free)).tw.
5 or/1-4
6 randomized controlled trial/
7 crossover procedure/
8 double-blind procedure/
9 random$.tw.
10 (crossover$ or cross-over$).tw.
11 placebo$.tw.
12 (doubl$ adj blind$).tw.
13 assign$.tw.
14 allocat$.tw.
15 or/6-14
16 (animal$ not (human$ and animal$)).mp.
17 15 not 16
18 5 and 17
19 remove duplicates from 18

Cochrane Central Register of Controlled Trials (Wiley)

----

1 MeSH descriptor Sodium, Chloride, Dietary
2 MeSH descriptor Sodium, Dietary explode all trees
3 MeSH descriptor Diet, Sodium-Restricted
4 sodium near3 (restrict* or curb* or limit* or minimi* or low* or reduc* or intake or diet* or free):ti,ab
5 salt near3 (restrict* or curb* or limit* or minimi* or low* or reduc* or intake or diet* or free):ti,ab
6 #1 or #2 or #3 or #4 or #5

Supplementary file 2 Study Characteristics

Study	Study country	No. of participants	Mean (range) age (years)	Female (%)	White (%)	Baseline SBP	Design	Study duration (days)	Change in UNa (mmol/24-hour)	Difference in change of BP (mmHg)	
										Systolic (SE)	Diastolic (SE)
Parijs 1973[1]	Belgium	15	41	55	NR	175	X	28	-98	-6.7 (3.48)	3.2 (4.24)
Mark 1975[2]	USA	6	27.8 (24-41)	0	NR	133	X	10	-305	-13.1 (2.77)	-7.0 (2.50)
Morgan 1981[3]	Australia	24	39	50	100	-	P	56	-88	-	-6.0 (2.41)
Skrabal 1981[4]	Austria	21	NR	0	NR	125	X	14	-170	-2.7 (2.36)	-3.0 (2.03)
MacGregor 1982[5]	UK	19	49 (30-66)	26	63	154	X	28	-76	-10.0 (2.40)	-5.0 (1.47)
Puska 1983[6]	Russia	72	NR	NR	100	153	P	42	-117	0.1 (3.23)	-0.7 (2.29)
Silman 1983[7]	UK	25	NR	NR	NR	155	P	365	-53	-8.7 (10.22)	-6.3 (4.42)
Watt 1983[8]	UK	18	52 (31-64)	67	100	137	X	28	-56	-0.5 (1.50)	-0.3 (0.80)
Erwteman 1984[9]	Netherlands	94	46	38	76	157	P	168	-58	-2.7 (2.20)	-3.4 (1.70)
Gillies 1984[10]	Australia	24	57	42	NR	147	X	42	-77	-2.4 (3.63)	-2.6 (2.46)
Koolen 1984[11]	Netherlands	25	41.3 (22-61)	40	100	148	X	14	-208	-6.2 (3.03)	-4.6 (1.87)
Koolen 1984[12]	Netherlands	20	40.8 (22-61)	45	100	148	X	14	-213	-6.5 (3.11)	-4.9 (1.71)
Maxwell 1984[13]	US	30	47	50	NR	149	P	7	-190	-6.3 (6.77)	-4.0 (4.24)
Myers 1984[14]	Australia	125	39	NR	NR	123	X	14	-147	-6.0 (1.00)	-4.0 (1.00)
Richards 1984[15]	New Zealand	12	NR	33	100	150	X	28	-105	-5.2 (4.10)	-1.8 (3.55)
Skrabal 1984[16]	Austria	52	NR	0	NR	120	X	14	-149	-2.4 (1.22)	-1.5 (0.99)
Resnick 1985[17]	US	12	NR	NR	NR	159	X	5	-200	-3.0 (1.89)	-1.0 (1.61)
Skrabal 1985[18]	Australia	62	NR	NR	NR	120	X	14	-153	-3.0 (0.65)	-1.5 (0.54)
Ashry 1987[19]	UK	26	26	38	100	129	X	14	-227	0.0 (2.28)	-2.5 (2.57)
Grobbee 1987[20]	Netherlands	40	24	15	NR	137	X	42	-72	-0.9 (1.80)	0.2 (1.67)
MacGregor 1987[21]	UK	15	52 (33-71)	27	67	150	X	30	-100	-13.0 (3.14)	-9.0 (3.02)
Morgan 1987[22]	Australia	20	60.5 (50-65)	0	NR	143	P	60	-62	-6.0 (5.00)	-4.2 (2.95)
Lawton 1988[23]	US	22	24.5 (20-31)	0	100	115	X	6	-319	-1.6 (2.04)	0.0 (1.47)
Morgan 1988[24]	Australia	16	63 (48-69)	0	NR	173	X	14	-50	-3.0 (1.41)	-4.0 (1.88)
Morgan 1988[25]	Australia	8	NR	NR	NR	156	X	14	-67	-7.0 (1.06)	-6.0 (1.06)
Nowson 1988[26]	Australia	107	52	15	100	150	P	84	-47	-5.1 (1.42)	-4.2 (0.85)
Nowson 1988[26]	Australia	105	52	15	100	149	P	84	-51	-0.2 (1.42)	0.5 (0.99)
Staessen 1988[27]	Belgium	1510	41	48	NR	131	P	1825	-12	-0.2 (1.73)	-1.0 (1.09)

Study	Study country	No. of participants	Mean (range) age (years)	Female (%)	White (%)	Baseline SBP	Design	Study duration (days)	Change in UNa (mmol/24-hour)	Difference in change of BP (mmHg)	
										Systolic (SE)	Diastolic (SE)
Chalmers 1989[28]	Australia	88	59	17	100	152	X	56	-67	-3.6 (0.70)	-2.1 (0.40)
Chalmers 1989[29]	Australia	108	59	17	100	144	P	56	-71	-5.5 (1.48)	-2.8 (0.85)
Dodson 1989[30]	UK	9	62	33	NR	171	X	30	-76	-9.7 (5.80)	-5.1 (2.94)
Hargreaves 1989[31]	Australia	8	23	0	NR	129	X	14	-106	-6.0 (4.00)	-3.0 (3.00)
MacGregor 1989[32]	UK	20	57 (42-72)	45	75	163	X	30	-141	-16.0 (4.12)	-9.0 (2.32)
Bruun 1990[33]	Denmark	22	46.5 (29-67)	36	NR	135	X	4	-336	-6.6 (4.08)	-2.6 (3.36)
Parker 1990[34]	Australia	59	52	0	NR	138	P	28	-93	1.0 (1.90)	0.4 (1.10)
Río 1990[35]	Spain	15	49.2 (36-65)	47	100	149	X	14	-100	-3.4 (2.76)	-1.1 (2.03)
Sharma 1990[36]	Germany	15	24 (20-31)	0	NR	107	X	7	-192	-0.9 (3.20)	-3.7 (2.69)
Sharma 1990[37]	Germany	40	25 (20-31)	0	NR	113	X	7	-214	-2.1 (1.43)	-3.1 (1.22)
Carney 1991[38]	Australia	11	54 (30-65)	55	NR	144	X	42	-102	-1.0 (5.57)	1.0 (3.61)
Creager 1991[39]	US	17	30	0	NR	122	X	5	-168	2.0 (3.00)	2.0 (2.00)
Sharma 1991[40]	Australia	23	24.9 (23-29)	0	NR	120	X	6	-246	-4.5 (1.43)	-2.3 (1.16)
Singer 1991[41]	UK	21	54	38	71	147	X	30	-91	-9.0 (2.34)	-3.0 (1.05)
Alli 1992[42]	Italy	56	48	57	NR	149	P	365	8	-6.3 (3.06)	-3.8 (1.32)
Arroll 1992[43]	New Zealand	181	55	48	NR	145	P	180	-1	-0.5 (2.50)	1.5 (1.50)
Benetos 1992[44]	France	20	42	55	100	149	X	28	-78	-6.5 (1.67)	-3.7 (1.29)
Cobiac 1992[45]	Australia	106	67	34	100	132	P	28	-71	-2.8 (1.41)	-1.3 (0.86)
Gow 1992[46]	UK	9	NR	0	NR	120	X	7	-94	-8.0 (3.47)	-3.0 (3.61)
Huggins 1992[47]	Australia	9	NR	22	NR	112	X	14	-97	-1.0 (2.00)	-2.0 (3.00)
Cutler 1992[48]	US	744	43	29	82	125	P	540	-44	-1.7 (0.59)	-0.9 (0.42)
Fotherby 1993[49]	UK	17	73 (66-79)	78	100	179	X	35	-79	-8.0 (3.77)	0.0 (2.39)
Nestel 1993[50]	Australia	66	66	45	100	146	P	42	-84	-3.9 (2.94)	-1.5 (2.25)
Redon-Mas 1993[51]	Spain	418	55	46	NR	163	P	28	-109	0.9 (1.41)	1.8 (0.92)
Río 1993[52]	Spain	30	49.2 (30-65)	43	NR	156	X	14	-151	-1.4 (2.26)	-0.5 (1.36)
Ruilope 1993[53]	Spain	19	NR	NR	NR	157	X	21	-67	-5.9 (3.35)	-5.3 (1.62)
Ruppert 1993[54]	Germany	163	38	40	NR	113	X	7	-274	-2.2 (0.84)	1.0 (0.51)
Sharma 1993[55]	Germany	16	NR	0	NR	111	X	7	-224	-1.4 (5.70)	-0.5 (1.67)
Sharma 1993[56]	Germany	15	25.3 (20-31)	0	NR	106	X	7	-174	-0.1 (2.13)	-2.1 (1.92)
Sharma 1993[57]	Germany	18	25.1 (21-28)	0	NR	111	X	7	-218	-1.8 (1.71)	-0.4 (1.42)

Study	Study country	No. of participants	Mean (range) age (years)	Female (%)	White (%)	Baseline SBP	Design	Study duration (days)	Change in UNa (mmol/24-hour)	Difference in change of BP (mmHg)	
										Systolic (SE)	Diastolic (SE)
Zoccali 1993[58]	Italy	14	47 (30-65)	0	NR	150	X	7	-169	-13.0 (2.71)	-6.0 (1.60)
Howe 1994[59]	Australia	56	55	45	NR	145	P	42	-78	-4.2 (2.09)	-1.5 (2.14)
Iwaoka 1994[60]	Japan	31	46	45	0	149	X	7	-266	-14.3 (3.92)	-4.6 (1.67)
MacFadyen 1994[61]	UK	12	NR	0	NR	114	X	3	-50	7.0 (2.71)	10.0 (2.16)
Zoccali 1994[62]	Italy	15	45 (30-65)	13	100	144	X	7	-163	-14.0 (4.70)	-8.0 (2.69)
Doig 1995[63]	US	8	25	0	NR	-	X	4	-112	-2.3 (1.73)	0.0 (2.01)
Draaijer 1995[64]	Netherlands	10	41	0	NR	159	X	7	-259	-7.5 (2.31)	-0.5 (0.22)
Stein 1995[65]	US	7	34	0	100	123	X	5	-183	1.4 (3.83)	-1.2 (3.10)
Weir 1995[66]	US	22	60	36	41	160	X	14	-136	-2.2 (2.75)	-1.4 (1.72)
Bellini 1996[67]	Italy	43	46	0	100	167	X	14	-217	-10.3 (2.67)	-9.8 (1.35)
Ferri 1996[68]	Italy	61	47	0	100	169	X	14	-265	-7.4 (1.78)	-3.5 (0.84)
Grey 1996[69]	New Zealand	34	23	0	100	116	X	7	-133	1.0 (1.74)	1.0 (1.07)
Inoue 1996[70]	UK	14	46 (21-59)	57	50	153	X	7	-293	-15.2 (2.62)	-3.7 (1.9)
Ishimitsu 1996[71]	Japan	30	54	53	0	147	X	7	-193	-11.8 (3.23)	-4.2 (1.78)
Schorr 1996[72]	Germany	16	64	56	100	134	X	28	-71	-7.2 (4.90)	-2.9 (2.61)
Zoccali 1996[73]	Italy	14	47 (37-59)	14	NR	140	X	7	-145	-11.0 (5.09)	-6.0 (2.78)
Cappuccio 1997[74]	UK	47	66.8 (60-78)	49	89	163	X	30	-83	-7.2 (3.02)	-3.2 (1.42)
Cutler 1997[75]	US	1190	44	33	80	127	P	1095	-40	-1.2 (0.50)	-0.7 (0.40)
McCarron 1997[76]	US	99	52	42	73	139	X	28	-55	-4.9 (1.87)	-2.9 (1.10)
Meland 1997[77]	Norway	16	50 (20-69)	19	100	145	X	56	-66	-4.0 (1.97)	-2.0 (1.25)
Schorr 1997[78]	Germany	90	25	0	NR	110	X	7	-196	0.1 (0.83)	0.8 (0.65)
Yamamoto 1997[79]	Japan	36	53.3 (40-69)	81	0	148	P	49	-32	-5.5 (6.40)	-3.3 (4.71)
Foo 1998[80]	UK	18	51	56	NR	127	X	6	-149	-5.8 (3.35)	2.0 (2.06)
Gomi 1998[81]	Japan	12	51.8 (38-65)	33	0	139	X	7	-70	-1.1 (2.22)	0.3 (1.16)
Herlitz 1998[82]	Sweden	6	NR	0	NR	129	X	4	-98	-5.0 (1.62)	-3.0 (0.97)
Wing 1998[83]	Australia	17	61 (37-74)	18	NR	160	X	42	-59	-7.0 (2.40)	-4.0 (1.37)
Davrath 1999[84]	US	8	25	0	NR	124	X	5	-96	8.0 (5.52)	5.0 (6.40)
Schorr 1999[85]	Germany	187	25.1 (20-30)	0	100	111	X	7	-207	-0.2 (0.63)	0.3 (0.65)
Uzu 1999[86]	Japan	70	50 (27-69)	33	0	153	X	7	-173	-15.4 (2.61)	-5.5 (1.37)
Boero 2000[87]	Italy	13	51 (21-64)	23	NR	132	X	14	-209	-4.0 (1.26)	-3.0 (0.94)

Study	Study country	No. of participants	Mean (range) age (years)	Female (%)	White (%)	Baseline SBP	Design	Study duration (days)	Change in UNa (mmol/24-hour)	Difference in change of BP (mmHg)	
										Systolic (SE)	Diastolic (SE)
Ames 2001[88]	US	21	60	52	62	154	X	28	-126	-6.0 (3.33)	-2.3 (1.71)
Appel 2001[89]	US	681	66	47	76	128	P	90	-40	-4.3 (0.89)	-2.0 (0.61)
Johnson 2001[90]	Australia	46	69	NR	NR	-	X	14	-237	-10.8 (2.51)	-5.9 (1.61)
Akita 2003[91]	US	375	48	57	39	129	X	30	-79	-5.0 (1.27)	-2.0 (0.51)
Dishy 2003[92]	US	25	34	40	84	112	X	6	-300	2.0 (7.81)	1.0 (1.88)
Nowson 2003[93]	Australia	92	45	63	NR	118	X	28	-88	0.4 (1.20)	0.0 (1.00)
Pechere-Bertschi 2003[94]	Switzerland	27	26 (20-40)	100	100	102	X	7	-296	-1.4 (2.34)	0.8 (1.48)
Perry 2003[95]	UK	15	26	0	NR	115	X	5	-105	0.0 (2.89)	-2.0 (2.50)
Beeks 2004[96]	Netherlands	117	53.6 (43-63)	43	NR	166	X	7	-99	1.2 (2.45)	-1.8 (1.06)
Berge-Landry 2004[97]	US	48	51	21	71	144	X	28	-285	-16.0 (4.56)	-8.0 (2.28)
Gates 2004[98]	US	12	64	50	100	144	X	28	-89	-7.0 (2.90)	-1.0 (1.83)
Forrester 2005[99]	Nigeria	58	47	41	0	122	X	21	-72	-4.8 (1.45)	-3.2 (1.00)
Forrester 2005[99]	Jamaica	56	41	39	0	114	X	21	-79	-5.1 (1.42)	-2.2 (1.45)
Swift 2005[100]	UK	40	50	58	0	159	X	28	-78	-8.0 (2.06)	-3.0 (1.11)
Cappuccio 2006[101]	Ghana	1013	55	62	0	128	P	180	6	-2.5 (2.04)	-4.0 (1.61)
Ho 2007[102]	Australia	25	49	68	NR	130	X	14	-210	-5.7 (1.50)	-2.5 (1.00)
Melander 2007[103]	Sweden	39	53	49	100	132	X	28	-89	-6.5 (1.50)	-3.3 (1.20)
Townsend 2007[104]	US	20	30	35	40	117	X	7	-171	-6.0 (4.10)	-4.0 (4.00)
Jessani 2008[105]	Pakistan	184	50	53	0	122	X	7	-81	-1.0 (0.77)	0.0 (0.77)
Tzemos 2008[106]	UK	16	27	0	100	121	X	5	-149	-4.0 (1.22)	-1.0 (1.45)
Visser 2008[107]	Netherlands	34	27	0	100	122	X	7	-181	-5.0 (2.46)	-1.0 (1.29)
Dickinson 2009[108]	Australia	29	53	76	NR	117	X	14	-92	-5.0 (2.03)	-1.0 (1.40)
He 2009[109]	UK	169	50	33	42	146	X	42	-55	-4.8 (0.82)	-2.2 (0.43)
Meland 2009[110]	Norway	46	56 (20-75)	26	NR	128	P	56	-38	-5.0 (2.73)	-5.0 (1.49)
Paulsen 2009[111]	UK	22	24 (22-30)	45	NR	111	X	4	-78	-1.0 (3.52)	1.0 (2.07)
Pimenta 2009[112]	US	12	56	67	50	146	X	7	-207	-22.7 (4.93)	-9.1 (2.73)
Weir 2010[113]	USA	115	52	45	86	134	X	28	-123	-9.4 (0.99)	-5.7 (0.64)
Zanchi 2010[114]	Switzerland	9	NR	0	NR	117	X	7	-250	-3.0 (7.94)	0.0 (4.58)
Starmans-Kool 2011[115]	UK	10	32 (22-40)	0	NR	114	X	14	-97	-2.0 (1.09)	0.0 (2.39)

Study	Study country	No. of participants	Mean (range) age (years)	Female (%)	White (%)	Baseline SBP	Design	Study duration (days)	Change in UNa (mmol/24-hour)	Difference in change of BP (mmHg)	
										Systolic (SE)	Diastolic (SE)
Carey 2012[116]	US	185	47.2 (18-70)	61	100	123	X	7	-203	-3.5 (1.05)	0.3 (0.65)
Carey 2012[116]	US, France	211	49	39	100	147	X	6	-212	-15.9 (1.25)	-9.2 (0.81)
Graffe 2012[117]	Denmark	21	26	52	100	110	X	4	-172	1.0 (3.36)	1.0 (1.92)
Bonfils 2013[118]	Denmark	36	40	58	NR	124	X	5	-140	-1.7 (2.57)	0.0 (1.30)
Mallamaci 2013[119]	Italy	32	48	28	NR	136	X	14	-165	-8.0 (2.20)	-3.0 (0.96)
Allen 2014[120]	US	70	24	63	NR	116	X	5	-306	0.0 (1.40)	3.0 (1.45)
Cavka 2015[121]	Croatia	54	20	100	NR	105	P	7	-141	-5.0 (2.91)	-3.0 (2.01)
Gijsbers 2015[122]	Netherlands	36	66	33	NR	137	X	28	-98	-7.5 (1.50)	-2.7 (0.76)
He 2015[123]	China	553	44	52	0	126	P	105	-50	-2.3 (1.16)	-0.9 (0.94)
Markota 2015[124]	Bosnia and Herzegovina	150	59	51	NR	175	P	60	-28	-4.9 (1.97)	-2.0 (0.60)
Matthews 2015[125]	US	20	41	50	70	120	X	7	-205	-5.0 (1.58)	-2.5 (1.21)
Riphagen 2016[126]	Netherlands	35	66	34	100	137	X	28	-99	-8.0 (1.50)	-2.9 (0.79)
Suckling 2016[127]	UK	46	58	48	70	136	X	42	-49	-4.3 (1.95)	-1.6 (1.15)
Brian 2017[128]	US	80	38	51	80	116	X	7	-287	-1.0 (0.78)	0.5 (0.71)
Gefke 2017[129]	Sweden	10	25 (22-30)	50	100	114	X	3	-169	-1.0 (2.00)	0.0 (0.88)
Babcock 2018[130]	US	21	38 (20-59)	50	NR	113	X	7	-177	0.0 (2.00)	3.0 (1.73)
Parvanova 2018[131]	Italy	115	64	11	NR	146	P	90	-44	-4.7 (2.02)	-3.0 (1.07)
Rorije 2018[132]	Italy	12	23 (18-31)	0	NR	118	X	8	-322	-1.0 (0.82)	0.0 (2.02)
Wang 2018[133]	China	90	51	64	0	122	X	7	-176	-9.6 (4.83)	-3.1 (1.56)

Abbreviations and symbols:

SBP: systolic blood pressure; Una: urinary sodium excretion; BP: blood pressure; SE: standard error; X: crossover design; P: parallel design

Supplementary file 3 Risk of bias assessment of included studies

Study	Sequence generation	Allocation concealment	Blinding of participants, personnel and outcome assessors	Incomplete outcome data	Selective reporting
Parijs 1973[1]	High risk (each patient received a number)	High risk (Those with uneven numbers were instructed to take a low-sodium diet during the first period and a high-sodium diet during the second period and vice versa for those with even numbers)	High risk (open study)	High risk (7 out of 22 participants were lost in the analysis)	Low risk
Mark 1975[2]	Unclear	Unclear	High risk (open study)	Low risk (no loss to follow up)	Low risk
Morgan 1981[3]	Unclear	Unclear	High risk (open study with BP observer blinded only)	Low risk (no loss to follow up)	High risk (SBP not reported)
Skrabal 1981[4]	Unclear	Unclear	High risk (open study)	Low risk (no loss to follow up)	Low risk
MacGregor 1982[5]	Unclear	Unclear	Low risk (double blinding)	Low risk	Low risk
Puska 1983[6]	Unclear	Unclear	High risk (open study with BP observer blinded only)	Low risk (4 out of 76 lost to follow up)	Low risk
Silman 1983[7]	Unclear	Unclear	High risk (open study with BP observer blinded only)	Low risk (no loss to follow up)	Low risk
Watt 1983[8]	Unclear	Unclear	Low risk (double blinding)	Low risk (2 out of 20 lost)	Low risk
Erwteman 1984[9]	Low risk (block randomization)	Unclear	High risk (Open study, with outcome observer blinded only)	Low risk (13 out of 107 lost to follow up)	Low risk
Gillies 1984[10]	Unclear	Unclear	High risk (open study)	Low risk (4 out of 28 lost to follow up)	Low risk
Koolen 1984[11]	Unclear	Unclear	High risk (Open study, with outcome observer blinded only)	Low risk (no loss to follow up)	Low risk
Koolen 1984[12]	Unclear	Unclear	High risk (open study)	Low risk (no loss to follow up)	Low risk
Maxwell 1984[13]	Unclear	Unclear	High risk (open study)	Low risk (no loss to follow up)	Low risk
Myers 1984[14]	Unclear	Unclear	Unclear	Low risk (ITT analysis)	Low risk
Richards 1984[15]	Unclear	Unclear	High risk (open study with BP measured by minicomputer)	High risk (4 out of 16 lost)	Low risk
Skrabal 1984[16]	Unclear	Unclear	High risk (open study)	Unclear	Low risk
Resnick 1985[17]	Unclear	Unclear	High risk (open study)	Low risk (no loss to follow up)	Low risk
Skrabal 1985[18]	Unclear	Unclear	High risk (open study with BP observer blinded only)	Low risk (no loss to follow up)	Low risk
Ashry 1987[19]	Unclear	Unclear	High risk (open study with BP observer blinded only)	Low risk (no loss to follow up)	Low risk
Grobbee 1987[20]	Unclear	Unclear	Low risk (double blinding)	Low risk (no loss to follow up)	Low risk
MacGregor 1987[21]	Unclear	Unclear	Low risk (double blinding)	Low risk (no loss to follow up)	Low risk

Study	Sequence generation	Allocation concealment	Blinding of participants, personnel and outcome assessors	Incomplete outcome data	Selective reporting
Morgan 1987[22]	Low risk (block randomization)	Unclear	High risk (open study with BP observer blinded only)	Low risk	Low risk
Lawton 1988[23]	Unclear	Unclear	High risk (open study)	Low risk (no loss to follow up)	Low risk
Morgan 1988[24]	Low risk (latin square)	Unclear	Low risk (double blinding)	Low risk (no loss to follow up)	Low risk
Morgan 1988[25]	Unclear	Unclear	Low risk (double blinding)	Unclear	Unclear
Nowson 1988[26]	Unclear	Unclear	High risk (open study with BP observer blinded only)	Low risk (ITT analysis)	Low risk
Nowson 1988[26]	Unclear	Unclear	High risk (open study with BP observer blinded only)	Low risk (ITT analysis)	Low risk
Staessen 1988[27]	Unclear	Unclear	High risk (Neither patients nor observers were blinded)	Low risk (<0.2 loss to follow up)	High risk
Chalmers 1989[28]	Unclear	Unclear	Low risk (double blinding)	Low risk (ITT analysis)	Low risk
Chalmers 1989[29]	Unclear	Unclear	Low risk (double blinding)	Low risk	Low risk
Dodson 1989[30]	Unclear	Unclear	Low risk (double blinding)	High risk (4 out of 13 lost to follow up)	High risk
Hargreaves 1989[31]	Unclear	Unclear	Low risk (double blinding)	Low risk (no loss to follow up)	Low risk
MacGregor 1989[32]	Unclear	Unclear	Low risk (double blinding)	Low risk (no loss to follow up)	Low risk
Bruun 1990[33]	Unclear	Unclear	High risk (open study)	Low risk (no loss to follow up)	Low risk
Parker 1990[34]	Unclear	Unclear	Low risk (double blinding)	Low risk (no loss to follow up)	Low risk
Río 1990[35]	Unclear	Unclear	Low risk (double blinding)	Low risk (no loss to follow up)	Low risk
Sharma 1990[36]	Unclear	Unclear	Low risk (participants blinded by placebo and BP was measured by automatic device)	Low risk (no loss to follow up)	Low risk
Sharma 1990[37]	Low risk (latin square design)	Unclear	High risk (open study with BP observer blinded only)	Low risk (5 out of total 45 was excluded from the analysis due to poor compliance)	High risk
Carney 1991[38]	Unclear	Unclear	Low risk (double blinding)	Low risk (no loss to follow up)	Low risk
Creager 1991[39]	Unclear	Unclear	Unclear	Low risk (no loss to follow up)	Low risk
Sharma 1991[40]	Unclear	Unclear	High risk (open study with BP observer blinded only)	Low risk (2 out of 25 lost to follow up)	Low risk
Singer 1991[41]	Unclear	Unclear	Low risk (double blinding)	Low risk (no loss to follow up)	Low risk
Alli 1992[42]	Unclear	Unclear	High risk (Neither patients nor observers were blinded)	High risk (21 out of 77 lost to follow up)	High risk

Study	Sequence generation	Allocation concealment	Blinding of participants, personnel and outcome assessors	Incomplete outcome data	Selective reporting
Arroll 1992[43]	Unclear	Unclear	High risk (open study with BP observer blinded only)	Low risk (27 out of 208 lost to follow up)	Low risk
Benetos 1992[44]	Low risk (computer randomization)	Unclear	Low risk (double blinding)	Low risk (2 out of 22 lost to follow up)	Low risk
Cobiac 1992[45]	Unclear	Unclear	Low risk (double blinding)	Low risk (1 out of 107 randomized was excluded from the analysis because the patient took antihypertensive medication, which was an exclution criteria of the study)	Low risk
Gow 1992[46]	Unclear	Unclear	High risk (open study)	Low risk (no loss to follow up)	Low risk
Huggins 1992[47]	Unclear	Unclear	Low risk (double blinding)	Low risk (no loss to follow up)	Low risk
Cutler 1992[48]	Low risk	Low risk (Randomization assignments were received from the coordinating center by telephone or sealed opaque envelopes were used to convey the treatment assignment)	High risk (open study with BP observer blinded only)	Low risk (ITT analysis)	Low risk
Fotherby 1993[49]	Unclear	Unclear	Low risk (double blinding)	Low risk (1 out of 18 lost to follow up)	Low risk
Nestel 1993[50]	Unclear	Unclear	Low risk (double blinding)	Low risk (no loss to follow up)	Low risk
Redon-Mas 1993[51]	Unclear	Unclear	High risk (open study)	High risk (156 out of 574 not included in the analysis because sodium excretion did not meet requirements of the study)	Low risk
Río 1993[52]	Unclear	Unclear	Low risk (double blinding)	High risk (17 out of 47 lost to follow up)	Low risk
Ruilope 1993[53]	Unclear	Unclear	Low risk (double blinding)	Low risk (no loss to follow up)	Low risk
Ruppert 1993[54]	Unclear	Unclear	Low risk (patients blinded by placebo and blood pressure measured by automated device)	Low risk (no loss to follow up)	Low risk
Sharma 1993[55]	Unclear	Unclear	High risk (open study with BP observer blinded only)	Low risk (no loss to follow up)	Low risk
Sharma 1993[56]	Low risk (randomized according to Latin square design)	Unclear	High risk (open study)	High risk (5 out of 20 lost)	Low risk
Sharma 1993[57]	Unclear	Unclear	High risk (open study with BP observer blinded only)	Low risk (no loss to follow up)	Low risk
Zoccali 1993[58]	Unclear	Unclear	High risk (open study with BP observer blinded only)	Low risk (no loss to follow up)	Low risk

Study	Sequence generation	Allocation concealment	Blinding of participants, personnel and outcome assessors	Incomplete outcome data	Selective reporting
Howe 1994[59]	Unclear	Unclear	Low risk (double blinding)	Low risk (5 out of 61 lost to follow up)	Low risk
Iwaoka 1994[60]	Unclear	Unclear	High risk (open study)	Low risk (no loss to follow up)	Low risk
MacFadyen 1994[61]	Unclear	Low risk (Treatments were administered in a randomised, double-blind, crossover design according to a pre-prepared schedule administered independently of the investigators by the Department of Pharmacy of the hospital)	Low risk (double blinding)	Low risk (no loss to follow up)	Low risk
Zoccali 1994[62]	Unclear	Unclear	High risk (open study with BP observer blinded only)	Low risk (no loss to follow up)	Low risk
Doig 1995[63]	Unclear	Low risk (randomization code prepared separately of the investigator)	Low risk (double blinding)	Low risk (no loss to follow up)	Low risk
Draaijer 1995[64]	Unclear	Unclear	High risk (open study with BP observer blinded only)	Low risk (no loss to follow up)	Low risk
Stein 1995[65]	Unclear	Unclear	High risk (open study)	Low risk (no loss to follow up)	Low risk
Weir 1995[66]	Unclear	Unclear	High risk (Patients blinded only, by using placebo)	Low risk (no loss to follow up)	Low risk
Bellini 1996[67]	Unclear	Unclear	Low risk (double blinding)	High risk (12 out of 55 lost to follow up)	High risk
Ferri 1996[68]	Unclear	Unclear	Low risk (double blinding)	Low risk (4 out of 65 lost to follow up)	Low risk
Grey 1996[69]	Unclear	Unclear	Low risk (double blinding)	Low risk (no loss to follow up)	Low risk
Inoue 1996[70]	Unclear	Unclear	Low risk (double blinding)	Low risk (no loss to follow up)	Low risk
Ishimitsu 1996[71]	Unclear	Unclear	High risk (open study)	Low risk (no loss to follow up)	Low risk
Schorr 1996[72]	Unclear	Unclear	Low risk (double blinding)	High risk (5 out of 16 lost to follow up)	Low risk
Zoccali 1996[73]	Unclear	High risk (study staff were aware of the allocation)	High risk (open study)	High risk (4 out of 18 lost to follow up)	Low risk
Cappuccio 1997[74]	Low risk (random-generated numbers handled by one not involved in the clinical assessments) in the clinical assessments)	Low risk (neither nurses nor participants were aware of the treatment allocation)	Low risk (double blinding)	Low risk (1 out of 48 lost to follow up)	Low risk
Cutler 1997[75]	Unclear	Low risk (Randomization was performed by telephone contact with the TOHP coordinating center or by opening a sealed opaque envelope)	High risk (open study with BP observer blinded only)	Unclear	Low risk

Study	Sequence generation	Allocation concealment	Blinding of participants, personnel and outcome assessors	Incomplete outcome data	Selective reporting
McCarron 1997[76]	Low risk (computer randomization)	Low risk (Placebo-controlled and study personnel blinded to the subjects' NaCl intake)	Low risk (double blinding)	Low risk (no loss to follow up)	Low risk
Meland 1997[77]	Unclear	Unclear	Low risk (double blinding)	Low risk (no loss to follow up)	Low risk
Schorr 1997[78]	Unclear	Unclear	High risk (Patients blinded only by using placebo)	Low risk (no loss to follow up)	Low risk
Yamamoto 1997[79]	Unclear	Unclear	Unclear	Low risk	High risk
Foo 1998[80]	Unclear	Unclear	Low risk (double blinding)	Low risk (no loss to follow up)	Low risk
Gomi 1998[81]	Unclear	Unclear	High risk (open study with BP observer blinded only)	Low risk (no loss to follow up)	Low risk
Herlitz 1998[82]	Unclear	Unclear	Low risk (double blinding)	Low risk (no loss to follow up)	Low risk
Wing 1998[83]	Low risk (latin square)	Unclear	Low risk (double blinding)	Low risk (2 out 19 lost)	Low risk
Davrath 1999[84]	Unclear	Unclear	High risk (open study with BP observer blinded only)	Low risk (no loss to follow up)	Low risk
Schorr 1999[85]	Unclear	Unclear	High risk (Open study, with outcome observer blinded only)	Low risk (no loss to follow up)	Low risk
Uzu 1999[86]	Unclear	Unclear	High risk (open study with BP observer blinded only)	Low risk (no loss to follow up)	Low risk
Boero 2000[87]	Unclear	Unclear	High risk (Intervention conducted by physicians and both the physicians and participants were aware of the allocation. Unclear if outcome observers were blinded or not)	Low risk (15 randomized, 1 withdrew consent, 1 didn't comply the protocol and 13 analyzed.)	Low risk
Ames 2001[88]	Unclear	Unclear	High risk (Patients blinded only, by using placebo)	High risk (13 out of 30 lost to follow up)	Low risk
Appel 2001[89]	Unclear	Unclear	High risk (open study with BP observer blinded only)	Low risk (ITT analysis)	Low risk
Johnson 2001[90]	Low risk (latin square)	Unclear	Low risk (double blinding)	Low risk (6 out of 46 lost)	Low risk
Akita 2003[91]	Unclear	Unclear	High risk (open study with BP observer blinded only)	Low risk (9% loss to follow up)	Low risk
Dishy 2003[92]	Unclear	Unclear	High risk (open study)	Low risk (no loss to follow up)	Low risk
Nowson 2003[93]	Low risk (random number in excel)	Low risk (Placebo-controlled and research personnel were unaware of the randomization status of subjects and tablet allocation)	Low risk (double blinding)	High risk (20 out of 128 lost to follow up and 16 hypertensive not reported)	High risk (results for hypertensive participants not reported)
Pechere-Bertschi 2003[94]	Unclear	Unclear	High risk (open study with BP observer blinded only)	Low risk (no loss to follow up)	Unclear

Study	Sequence generation	Allocation concealment	Blinding of participants, personnel and outcome assessors	Incomplete outcome data	Selective reporting
Perry 2003[95]	Unclear	Unclear	Low risk (double blinding)	Low risk (no loss to follow up)	Low risk
Beeks 2004[96]	Unclear	Unclear	High risk (open study)	Low risk (no loss to follow up)	Low risk
Berge-Landry 2004[97]	Unclear	Unclear	High risk (open study)	Low risk (no loss to follow up)	Low risk
Gates 2004[98]	Unclear	Unclear	Low risk (double blinding)	Low risk (no loss to follow up)	Low risk
Forrester 2005[99]	Low risk (block randomization)	Unclear	High risk (open study)	Low risk (No loss to follow up)	Low risk
Forrester 2005[99]	Low risk (block randomization)	Unclear	Unclear	Low risk (No loss to follow up)	Low risk
Swift 2005[100]	Unclear	Unclear	Low risk (double blinding)	Low risk (6 out of 46 lost to follow up)	Low risk
Cappuccio 2006[101]	Low risk (Villages were randomised in blocks of two, and stratified for locality (semi-urban or rural) by an independent statistician)	Low risk	High risk (Community health workers conducted the intervention and was not possible to be blinded. Only participants were blinded. Unclear if outcome assessors were blinded or not)	Low risk (ITT analysis)	Low risk
Ho 2007[102]	Unclear	Unclear	High risk (open study with BP observer blinded only)	High risk (19 out of 44 lost to follwow up)	Low risk
Melander 2007[103]	Unclear	Unclear	Low risk (double blinding)	Low risk (7 out of 46 lost to follow up)	Low risk
Townsend 2007[104]	Low risk (using a pre-specified randomized blocked (block=ten subjects) table generated by the GCRC biostatistician and kept by the dietician)	Low risk (using a pre-specified randomized blocked (block=ten subjects) table generated by the GCRC biostatistician and kept by the dietician)	Low risk (double blinding)	Low risk (no loss to follow up)	Low risk
Jessani 2008[105]	Low risk (computer randomization)	Low risk	High risk (open study with BP observer blinded only)	Low risk (16 out of 200 lost to follow up)	Low risk
Tzemos 2008[106]	Unclear	Unclear	Low risk (double blinding)	Low risk (no loss to follow up)	Low risk
Visser 2008[107]	Unclear	Unclear	Low risk (double blinding)	Low risk (no loss to follow up)	Unclear
Dickinson 2009[108]	Low risk (computer randomization)	Unclear	High risk (open study with BP observer blinded only)	Low risk (2 out of 31 lost to follow up)	Low risk
He 2009[109]	Low risk (computer randomization)	Low risk	Low risk (double blinding)	Low risk (16 out of 185 lost to follow up)	Low risk
Meland 2009[110]	Unclear (simple randomization to ensure equal number in both groups, but didn't	Low risk (The randomization list was concealed and kept inaccessible to the investigators during the trial, and was	Low risk (double blinding)	Low risk (4 out of 50 lost to follow up)	Low risk

Study	Sequence generation	Allocation concealment	Blinding of participants, personnel and outcome assessors	Incomplete outcome data	Selective reporting
	mention how it was done)	disclosed after all the statistical analyses had been conducted)			
Paulsen 2009[111]	Unclear	Low risk (randomization and blinding of sodium chloride tablets was conducted by the hospital pharmacy)	Low risk (double blinding)	Low risk (5 out of 27 lost to follow up)	Low risk
Pimenta 2009[112]	Unclear	Unclear	High risk (open study)	Low risk (1 out of 13 lost to follow up)	Low risk
Weir 2010[113]	Unclear	Unclear	High risk (open study with BP observer blinded only)	Low risk (17 out of 132 lost to follow up)	Low risk
Zanchi 2010[114]	Unclear	Unclear	Low risk (double blinding)	Low risk (1 out of 10 lost to follow up)	Low risk
Starmans-Kool 2011[115]	Low risk (computer randomization)	Low risk (A copy of the list was given to the hospital kitchen, and the original was kept in a sealed envelope at the department. The code was revealed when the study was finished)	Low risk (double blinding)	Low risk (4 out of 25 lost to follow up)	Low risk
Carey 2012[116]	Unclear	Unclear	Low risk (double blinding)	Low risk (no loss to follow up)	Low risk
Carey 2012[116]	Unclear	Unclear	High risk (open study)	Unclear	Low risk
Graffe 2012[117]	Unclear	Unclear	High risk (open study)	Unclear	Low risk
Bonfils 2013[118]	Low risk (tossing coin)	High risk (The primary investigator assigned participants to the interventions according to the randomization sequence. The patients were not blinded for treatment assignment)	High risk (open study with BP observer blinded only)	Low risk (ITT analysis)	Low risk
Mallamaci 2013[119]	Unclear	Unclear	Low risk (intervention was placebo controlled and 24-hour ambulatory BP was measured with a device)	Low risk (no loss to follow up)	Low risk
Allen 2014[120]	Unclear	Unclear	High risk (open study with BP observer blinded only)	Unclear (No. randomized not reported and no loss to follow up was reported)	Unclear
Cavka 2015[121]	Unclear	Unclear	High risk (Patients blinded only, by using placebo)	Low risk (no loss to follow up)	Low risk
Gijsbers 2015[122]	Low risk (computer-generated table)	Low risk (independent person)	Low risk (double blinding)	Low risk (1 out of 37 lost to follow up)	Low risk
He 2015[123]	Low risk (random number list generated by a researcher who was blind to the identity of the participants)	Low risk	High risk (open study with BP observer blinded only)	Low risk (ITT analysis)	Low risk

Study	Sequence generation	Allocation concealment	Blinding of participants, personnel and outcome assessors	Incomplete outcome data	Selective reporting
Markota 2015[124]	Unclear	Low risk (sealed envelope)	High risk (open study)	Low risk (no loss to follow up)	Low risk
Matthews 2015[125]	Unclear	Unclear	Unclear	Low risk (no loss to follow up)	High risk (results only reported for 20 out of 41)
Riphagen 2016[126]	Unclear	Unclear	Low risk (double blinding)	Low risk (1 out of 36 lost to follow up)	Low risk
Suckling 2016[127]	Low risk (computer randomization)	Low risk (independent company)	Low risk (double blinding)	Low risk (3 out of 49 lost to follow up)	Low risk
Brian 2017[128]	Unclear	Unclear	High risk (open study with patients not blinded to assignment)	High risk (21 out of 101 lost to follow up)	High risk
Gefke 2017[129]	Unclear	Unclear	High risk (open study)	Low risk	Low risk
Babcock 2018[130]	Unclear	Unclear	High risk (open study with BP observer blinded only)	Low risk	Low risk
Parvanova 2018[131]	Low risk	Low risk	High risk (open study with BP observer blinded only)	Low risk	Low risk
Rorije 2018[132]	Low risk (block randomization)	Unclear	High risk (open study)	Low risk	Low risk
Wang 2018[133]	Unclear	Unclear	High risk (open study with BP observer blinded only)	Low risk	Low risk

References

1. Parijs J, Joossens J, Linden L, et al. Moderate sodium restriction and diuretics in the treatment of hypertension. *Am Heart J.* 1973;85(1):22-34.
2. Mark A, Lawton W, Abboud F, et al. Effects of high and low sodium intake on arterial pressure and forearm vascular resistance in borderline hypertension. A preliminary report. *Circulation Research.* 1975;36(6 Suppl 1):194-8.
3. Morgan T, Myers J. Hypertension treated by sodium restriction. *Medical journal of Australia.* 1981;2(8):396-7.
4. Skrabal F, Auböck J, Hörtnagl H. Low sodium/high potassium diet for prevention of hypertension: probable mechanisms of action. *Lancet.* 1981;2(8252):895-900.
5. MacGregor G, Markandu N, Best F, et al. Double-blind randomised crossover trial of moderate sodium restriction in essential hypertension. *Lancet.* 1982;1(8268):351-5.
6. Puska P, Iacono J, Nissinen A, et al. Controlled, randomised trial of the effect of dietary fat on blood pressure. *Lancet.* 1983;1(8314-5):1-5.
7. Silman AJ, Mitchell P, Locke C, et al. Evaluation of the effectiveness of a low sodium diet in the treatment of mild to moderate hypertension. *Lancet.* 1983;1(8335):1179-82.
8. Watt GCM, Edwards C, Hart JT. Dietary sodium restriction for mild hypertension in general practice. *BMJ.* 1983;286(6363):432-6.
9. Erwteman TM, Nagelkerke N, Lubsen J, et al. beta Blockade, diuretics, and salt restriction for the management of mild hypertension: A randomised double blind trial. *BMJ.* 1984;289(6442):406-9.
10. Gillies A, Carney S, Smith A, et al. Adjunctive effect of salt restriction on antihypertensive efficacy. *Clin Exp Pharmacol Physiol.* 1984;11(4):395-8.
11. Koolen M, Brummelen P. Adrenergic activity and peripheral hemodynamics in relation to sodium sensitivity in patients with essential hypertension. *Hypertension.* 1984;6(6 Pt 1):820-5.
12. Koolen M, Brummelen P. Sodium sensitivity in essential hypertension: role of the renin-angiotensin-aldosterone system and predictive value of an intravenous frusemide test. *J Hypetens.* 1984;2(1):55-9.
13. Maxwell M, Kushiro T, Dornfeld L, et al. BP changes in obese hypertensive subjects during rapid weight loss. Comparison of restricted v unchanged salt intake. *Arch Intern Med.* 1984;144(8):1581-4.
14. Myers J, Morgan T. Effect of alteration in sodium chloride intake on blood pressure of normotensive subjects. *J Cardiovasc Pharmacol.* 1984;6 Suppl 1:S204-9.
15. Richards A, Espiner E, Maslowski A, et al. Blood pressure response to moderate sodium restriction and to potassium supplementation in mild essential hypertension. *Lancet.* 1984;I(8380):757-61.
16. Skrabal F, Herholz H, Neumayr M, et al. Salt sensitivity in humans is linked to enhanced sympathetic responsiveness and to enhanced proximal tubular reabsorption. *Hypertension.* 1984;6(2 Pt 1):152-8.
17. Resnick L, Nicholson J, Laragh J. Alterations in calcium metabolism mediate dietary salt sensitivity in essential hypertension. *Trans Assoc Am Physicians.* 1985;98:313-21.
18. Skrabal F, Hamberger L, Cerny E. Salt sensitivity in normotensives with and salt resistance in normotensives without heredity of hypertension. *Scand J Clin Lab Invest.* 1985;45(Suppl. 176):47-57.
19. Ashry A, Heagerty A, Alton S, et al. Effects of manipulation of sodium balance on erythrocyte sodium transport. *J Hum Hypertens.* 1987;1(2):105-11.
20. Grobbee D, Hofman A, Roelandt J, et al. Sodium restriction and potassium supplementation in young people with mildly elevated blood pressure. *J Hypetens.* 1987;5(1):115-9.
21. MacGregor GA, Markandu ND, Singer DRJ. Moderate sodium restriction with angiotensin converting enzyme inhibitor in essential hypertension: A double blind study. *BMJ.* 1987;294(6571):531-4.
22. Morgan T, Anderson A. Sodium restriction can delay the return of hypertension in patients previously well-controlled on drug therapy. *Can J Physiol Pharmacol.* 1987;65(8):1752-5.
23. Lawton W, Sinkey C, Fitz A, et al. Dietary salt produces abnormal renal vasoconstrictor responses to upright posture in borderline hypertensive subjects. *Hypertension.* 1988;11(6 Pt 1):529-36.
24. Morgan T, Anderson A. Interaction in hypertensive man between sodium intake, converting enzyme inhibitor (enalapril), plasma renin and blood pressure control. *J Hum Hypertens.* 1988;1(4):311-5.
25. Morgan T, Anderson A. Interaction of slow-channel calcium blocking drugs with sodium restriction, diuretics and angiotensin converting enzyme inhibitors. *J Hypetens.* 1988;6 (puppl 4):S652-4.
26. Nowson CA, Morgan TO. Change in blood pressure in relation to change in nutrients effected by manipulation of dietary sodium and potassium. *Clin Exp Pharmacol Physiol.* 1988;15(3):225-42.

27. Staessen J, Bulpitt C, Fagard R, et al. Salt intake and blood pressure in the general population: a controlled intervention trial in two towns. *J Hypetens.* 1988;6(12):965-73.
28. Chalmers JP, Doyle AE, Hopper JL, et al. Effects of replacing sodium intake in subjects on a low sodium diet: A crossover study. *Clin Exp Hypertens A.* 1989;11(5-6):1011-24.
29. Chalmers JP, Doyle AE, Hopper JL, et al. Fall in blood pressure with modest reduction in dietary salt intake in mild hypertension. *Lancet.* 1989;1(8635):399-402.
30. Dodson PM, Beevers M, Hallworth R, et al. Sodium restriction and blood pressure in hypertensive type II diabetics: Randomised blind controlled and crossover studies of moderate sodium restriction and sodium supplementation. *BMJ.* 1989;298(6668):227-30.
31. Hargreaves M, Morgan T, Snow R, et al. Exercise tolerance in the heat on low and normal salt intakes. *Clin Sci.* 1989;76(5):553-7.
32. MacGregor GA, Markandu ND, Sagnella GA, et al. Double-blind study of three sodium intakes and long-term effects of sodium restriction in essential hypertension. *Lancet.* 1989;2(8674):1244-7.
33. Bruun N, Skøtt P, Nielsen M, et al. Normal renal tubular response to changes of sodium intake in hypertensive man. *J Hypetens.* 1990;8:219-27.
34. Parker M, Puddey IB, Beilin LJ, et al. Two-way factorial study of alcohol and salt restriction in treated hypertensive men. *Hypertension.* 1990;16(4):398-406.
35. Río A, Rodríguez-Villamil J, López-Campos J, et al. Effect of moderate salt restriction on the antihypertensive action of nifedipine: a double blind study. *Rev Clin Esp.* 1990;186(1):5-10.
36. Sharma AM, Arntz HR, Kribben A, et al. Dietary sodium restriction: Adverse effect on plasma lipids. *Klinische Wochenschrift.* 1990;68(13):664-8.
37. Sharma AM, Kribben A, Schattenfroh S, et al. Salt sensitivity in humans is associated with abnormal acid-base regulation. *Hypertension.* 1990;16(4):407-13.
38. Carney S, Gillies A, Smith A, et al. Increased dietary sodium chloride in patients treated with antihypertensive drugs. *Clin Exp Hypertens A.* 1991;13(3):401-7.
39. Creager M, Roddy M, Holland K, et al. Sodium depresses arterial baroreceptor reflex function in normotensive humans. *Hypertension.* 1991;17(6 Pt 2):989-96.
40. Sharma A, Ruland K, Spies K, et al. Salt sensitivity in young normotensive subjects is associated with a hyperinsulinemic response to oral glucose. *J Hypetens.* 1991;9(4):329-35.
41. Singer DRJ, Markandu ND, Sugden AL, et al. Sodium restriction in hypertensive patients treated with a converting enzyme inhibitor and a thiazide. *Hypertension.* 1991;17(6 I):798-803.
42. Alli C, Avanzini F, Bettelli G, et al. Feasibility of a long-term low-sodium diet in mild hypertension. *J Hum Hypertens.* 1992;6(4):281-6.
43. Arroll B. The Auckland blood pressure control study: a randomised controlled trial of physical activity and salt restriction in persons. *Medical and health sciences.* 1992;PhD.
44. Benetos A, Yang-Yan X, Cuche JL, et al. Arterial effects of salt restriction in hypertensive patients. A 9-week, randomized, double-blind, crossover study. *J Hypetens.* 1992;10(4):355-60.
45. Cobiac L, Nestel PJ, Wing LMH, et al. A low-sodium diet supplemented with fish oil lowers blood pressure in the elderly. *J Hypetens.* 1992;10(1):87-92.
46. Gow I, Dockrell M, Edwards C, et al. The sensitivity of human blood platelets to the aggregating agent ADP during different dietary sodium intakes in healthy men. *Eur J Clin Pharmacol.* 1992;43(6):635-8.
47. Huggins R, Nicolantonio R, Morgan T. Preferred salt levels and salt taste acuity in human subjects after ingestion of untasted salt. *Appetite.* 1992;18(2):111-9.
48. Cutler JA, Whelton PK, Appel L, et al. The effects of nonpharmacologic interventions on blood pressure of persons with high normal levels: Results of the trials of hypertension prevention, phase I. *JAMA.* 1992;267(9):1213-20.
49. Fotherby MD, Potter JF. Effects of moderate sodium restriction on clinic and twenty-four-hour ambulatory blood pressure in elderly hypertensive subjects. *J Hypetens.* 1993;11(6):657-63.
50. Nestel PJ, Clifton PM, Noakes M, et al. Enhanced blood pressure response to dietary salt in elderly women, especially those with small waist: Hip ratio. *J Hypetens.* 1993;11(12):1387-94.
51. Redon-Mas J, Abellan-Aleman J, Aranda-Lara P, et al. Antihypertensive activity of verapamil: Impact of dietary sodium. *J Hypetens.* 1993;11(6):665-71.
52. Río A, Rodríguez-Villamil J. Metabolic effects of strict salt restriction in essential hypertensive patients. *J Intern Med.* 1993;233(5):409-14.

53. Ruilope LM, Lahera V. Influence of salt intake on the antihypertensive effect of carvedilol. *J Hypetens.* 1993;11(SUPPL. 4):S17-S9.
54. Ruppert M, Overlack A, Kolloch R, et al. Neurohormonal and metabolic effects of severe and moderate salt restriction in non-obese normotensive adults. *J Hypetens.* 1993;11(7):743-9.
55. Sharma AM, Schorr U, Thiede HM, et al. Effect of dietary salt restriction on urinary serotonin and 5-hydroxyindoleacetic acid excretion in man. *J Hypetens.* 1993;11(12):1381-6.
56. Sharma A, Schorr U, Oelkers W, et al. Effects of sodium salts on plasma renin activity and norepinephrine response to orthostasis in salt-sensitive normotensive subjects. *Am J Hypertens.* 1993;6(9):780-5.
57. Sharma AM, Schorr U, Distler A. Insulin resistance in young salt-sensitive normotensive subjects. *Hypertension.* 1993;21(3):273-9.
58. Zoccali C, Mallamaci F, Leonardis D, et al. Randomly allocated crossover study of various levels of sodium intake in patients with mild hypertension. *J Hypetens.* 1993;11(suppl 5):S326-7.
59. Howe PRC, Lungershausen YK, Cobiac L, et al. Effect of sodium restriction and fish oil supplementation on BP and thrombotic risk factors in patients treated with ACE inhibitors. *J Hum Hypertens.* 1994;8(1):43-9.
60. Iwaoka T, Umeda T, Inoue J, et al. Dietary NaCl restriction deteriorates oral glucose tolerance in hypertensive patients with impairment of glucose tolerance. *Am J Hypertens.* 1994;7(5):460-3.
61. MacFadyen RJ, Lees KR, Reid JL. Responses to low dose intravenous perindoprilat infusion in salt deplete/salt replete normotensive volunteers. *Br J clin Pharmac.* 1994;38(4):329-34.
62. Zoccali C, Mallamaci F, Parlongo S. The influence of salt intake on plasma calcitonin gene-related peptide in subjects with mild essential hypertension. *J Hypetens.* 1994;12(11):1249-53.
63. Doig J, MacFadyen R, Sweet C, et al. Haemodynamic and renal responses to oral losartan potassium during salt depletion or salt repletion in normal human volunteers. *J Cardiovasc Pharmacol.* 1995;25(4):511-7.
64. Draaijer P, De Leeuw P, Maessen J, et al. Salt-sensitivity testing in patients with borderline hypertension: Reproducibility and potential mechanisms. *J Hum Hypertens.* 1995;9(4):263-9.
65. Stein CM, Nelson R, Brown M, et al. Dietary sodium intake modulates systemic but not forearm norepinephrine release. *Clin Pharmacol Ther.* 1995;58(4):425-33.
66. Weir MR, Dengel DR, Behrens MT, et al. Salt-induced increases in systolic blood pressure affect renal hemodynamics and proteinuria. *Hypertension.* 1995;25(6):1339-44.
67. Bellini C, Ferri C, Carlomagno A, et al. Impaired inactive to active kallikrein conversion in human salt-sensitive hypertension. *J Am Soc Nephrol.* 1996;7(12):2565-77.
68. Ferri C, Bellini C, Carlomagno A, et al. Active kallikrein respons to changes in sodium-chloride intake in essential hypertensive patients. *J Am Soc Nephrol.* 1996;7(3):443-53.
69. Grey A, Braatvedt G, Holdaway I. Moderate dietary salt restriction does not alter insulin resistance or serum lipids in normal men. *Am J Hypertens.* 1996;9(4 Pt 1):317-22.
70. Inoue J, Cappuccio F, Sagnella G, et al. Glucose load and renal sodium handling in mild essential hypertension on different sodium intakes. *J Hum Hypertens.* 1996;10(8):523-9.
71. Ishimitsu T, Nishikimi T, Matsuoka H, et al. Behaviour of adrenomedullin during acute and chronic salt loading in normotensive and hypertensive subjects. *Clin Sci.* 1996;91(3):293-8.
72. Schorr U, Distler A, Sharma AM. Effect of sodium chloride- and sodium bicarbonate-rich mineral water on blood pressure and metabolic parameters in elderly normotensive individuals: A randomized double-blind crossover trial. *J Hypetens.* 1996;14(1):131-5.
73. Zoccali C, Mallamaci F, Cuzzola F, et al. Reproducibility of the response to short-term low salt intake in essential hypertension. *J Hypetens.* 1996;14(12):1455-9.
74. Cappuccio FP, Markandu ND, Carney C, et al. Double-blind randomised trial of modest salt restriction in older people. *Lancet.* 1997;350(9081):850-4.
75. Cutler JA. Effects of weight loss and sodium reduction intervention on blood pressure and hypertension incidence in overweight people with high-normal blood pressure: The trials of hypertension prevention, phase II. *Arch Intern Med.* 1997;157(6):657-67.
76. McCarron DA, Weder AB, Egan BM, et al. Blood pressure and metabolic responses to moderate sodium restriction in isradipine-treated hypertensive patients. *Am J Hypertens.* 1997;10(1):68-76.
77. Meland E, Laerum E, Aakvaag A, et al. Salt restriction: effects on lipids and insulin production in hypertensive patients. *Scand J Clin Lab Invest.* 1997;57(6):501-5.

78. Schorr U, Turan S, Distler A, et al. Relationship between ambulatory and resting blood pressure responses to dietary salt restriction in normotensive men. *J Hypetens.* 1997;15(8):845-9.
79. Yamamoto H. Randomized controlled trial of salt-restriction program for primary prevention of hypertension in the community. *Journal of the osaka city medical center.* 1997;46(3-4):255-67.
80. Foo M, Denver A, Coppack S, et al. Effect of salt-loading on blood pressure, insulin sensitivity and limb blood flow in normal subjects. *Clin Sci.* 1998;95(2):157-64.
81. Gomi T, Shibuya Y, Sakurai J, et al. Strict dietary sodium reduction worsens insulin sensitivity by increasing sympathetic nervous activity in patients with primary hypertension. *Am J Hypertens.* 1998;11(9):1048-55.
82. Herlitz H, Dahlöf B, Jonsson O, et al. Relationship between salt and blood pressure in hypertensive patients on chronic ACE-inhibition. *Blood pressure.* 1998;7(1):47-52.
83. Wing L, Arnolda L, Harvey P, et al. Low-dose diuretic and/or dietary sodium restrication when blood pressure is resistant to ACE inhibitor. *Blood pressure.* 1998;7(5-6):299-307.
84. Davrath L, Gotshall R, Tucker A, et al. Moderate sodium restriction does not alter lower body negative pressure tolerance. *Aviat Space Environ Med.* 1999;70(6):577-82.
85. Schorr U, Blaschke K, Beige J, et al. Angiotensinogen M235T variant and salt sensitivity in young normotensive Caucasians. *J Hypetens.* 1999;17(4):475-9.
86. Uzu T, Fujii T, Nishimura M, et al. Determinants of circadian blood pressure rhythm in essential hypertension. *Am J Hypertens.* 1999;12(1 Pt 1):35-9.
87. Boero R, Pignataro A, Bancale E, et al. Metabolic effects of changes in dietary sodium intake in patients with essential hypertension. [Italian]. *Minerva Urol Nefrol.* 2000;52(1):13-6.
88. Ames RP. The effect of sodium supplementation on glucose tolerance and insulin concentrations in patients with hypertension and diabetes mellitus. *Am J Hypertens.* 2001;14(7 I):653-9.
89. Appel L, Espeland M, Easter L, et al. Effects of reduced sodium intake on hypertension control in older individuals: results from the Trial of Nonpharmacologic Interventions in the Elderly (TONE). *Arch Intern Med.* 2001;161(5):685-93.
90. Johnson AG, Nguyen TV, Davis D. Blood pressure is linked to salt intake and modulated by the angiotensinogen gene in normotensive and hypertensive elderly subjects. *J Hypetens.* 2001;19(6):1053-60.
91. Akita S, Sacks F, Svetkey L, et al. Effects of the Dietary Approaches to Stop Hypertension (DASH) diet on the pressure-natriuresis relationship. *Hypertension.* 2003;42(1):8-13.
92. Dishy V, Sofowora G, Imamura H, et al. Nitric oxide production decreases after salt loading but is not related to blood pressure changes or nitric oxide-mediated vascular responses. *J Hypetens.* 2003;21(1):153-7.
93. Nowson CA, Morgan TO, Gibbons C. Decreasing Dietary Sodium while Following a Self-Selected Potassium-Rich Diet Reduces Blood Pressure. *J Nutr.* 2003;133(12):4118-23.
94. Pechere-Bertschi A, Maillard M, Stalder H, et al. Renal hemodynamic and tubular responses to salt in women using oral contraceptives. *Kidney International.* 2003;64(4):1374-80.
95. Perry C, Palmer T, Cleland S, et al. Decreased insulin sensitivity during dietary sodium restriction is not mediated by effects of angiotensin II on insulin action. *Clin Sci.* 2003;105(2):187-94.
96. Beeks E, Klauw M, Kroon A, et al. Alpha-adducin Gly460Trp polymorphism and renal hemodynamics in essential hypertension. *Hypertension.* 2004;44(4):419-23.
97. Berge-Landry H, James G. Serum electrolyte, serum protein, serum fat and renal responses to a dietary sodium challenge: allostasis and allostatic load. *Ann Hum Biol.* 2004;31(4):477-87.
98. Gates P, Tanaka H, Hiatt W, et al. Dietary sodium restriction rapidly improves large elastic artery compliance in older adults with systolic hypertension. *Hypertension.* 2004;44(1):35-41.
99. Forrester T, Adeyemo A, Soarres-Wynter S, et al. A randomized trial on sodium reduction in two developing countries. *J Hum Hypertens.* 2005;19(1):55-60.
100. Swift PA, Markandu ND, Sagnella GA, et al. Modest salt reduction reduces blood pressure and urine protein excretion in black hypertensives: A randomized control trial. *Hypertension.* 2005;46(2):308-12.
101. Cappuccio F, Kerry S, Micah F, et al. A community programme to reduce salt intake and blood pressure in Ghana. *BMC public health.* 2006;6:13.
102. Ho J, Keogh J, Bornstein S, et al. Moderate weight loss reduces renin and aldosterone but does not influence basal or stimulated pituitary-adrenal axis function. *Horm Metab Res.* 2007;39(9):694-9.

103. Melander O, Wowern F, Frandsen E, et al. Moderate salt restriction effectively lowers blood pressure and degree of salt sensitivity is related to baseline concentration of renin and N-terminal atrial natriuretic peptide in plasma. *J Hypetens.* 2007;25(3):619-27.
104. Townsend R, Kapoor S, McFadden C. Salt intake and insulin sensitivity in healthy human volunteers. *Clin Sci.* 2007;113(3):141-8.
105. Jessani S, Hatcher J, Chaturvedi N, et al. Effect of low vs. high dietary sodium on blood pressure levels in a normotensive Indo-Asian population. *Am J Hypertens.* 2008;21(11):1238-44.
106. Tzemos N, Lim P, Wong S, et al. Adverse cardiovascular effects of acute salt loading in young normotensive individuals. *Hypertension.* 2008;51(6):1525-30.
107. Visser F, Boonstra A, Titia LA, et al. Renal response to angiotensin II is blunted in sodium-sensitive normotensive men. *Am J Hypertens.* 2008;21(3):323-8.
108. Dickinson K, Keogh J, Clifton P. Effects of a low-salt diet on flow-mediated dilatation in humans. *Am J Clin Nutr.* 2009;89(2):485-90.
109. He F, Marciniak M, Visagie E, et al. Effect of modest salt reduction on blood pressure, urinary albumin, and pulse wave velocity in white, black, and Asian mild hypertensives. *Hypertension.* 2009;54(3):482-8.
110. Meland E, Aamland A. Salt restriction among hypertensive patients: modest blood pressure effect and no adverse effects. *Scand J Prim Health Care.* 2009;27(2):97-103.
111. Paulsen L, Holst L, Bech J, et al. Glomerular filtration rate and blood pressure are unchanged by increased sodium intake in atorvastatin-treated healthy men. *Scand J Clin Lab Invest.* 2009;69(3):323-9.
112. Pimenta E, Gaddam KK, Oparil S, et al. Effects of dietary sodium reduction on blood pressure in subjects with resistant hypertension: Results from a randomized trial. *Hypertension.* 2009;54(3):475-81.
113. Weir MR, Yadao AM, Purkayastha D, et al. Effects of high- and low-sodium diets on ambulatory blood pressure in patients with hypertension receiving aliskiren. *J Cardiovasc Pharmacol Ther.* 2010;15(4):356-63.
114. Zanchi A, Maillard M, Jornayvaz F, et al. Effects of the peroxisome proliferator-activated receptor (PPAR)-gamma agonist pioglitazone on renal and hormonal responses to salt in diabetic and hypertensive individuals. *Diabetologia.* 2010;53(8):1568-75.
115. Starmans-Kool MJ, Stanton AV, Xu YY, et al. High dietary salt intake increases carotid blood pressure and wave reflection in normotensive healthy young men. *J Appl Physiol.* 2011;110(2):468-71.
116. Carey RM, Schoeffel CD, Gildea JJ, et al. Salt sensitivity of blood pressure is associated with polymorphisms in the sodium-bicarbonate cotransporter. *Hypertension.* 2012;60(5):1359-66.
117. Graffe CC, Bech JN, Pedersen EB. Effect of high and low sodium intake on urinary aquaporin-2 excretion in healthy humans. *Am J Physiol Renal Physiol.* 2012;302(2):F264-F75.
118. Bonfils P, Taskiran M, Damgaard M, et al. The influence of high versus low sodium intake on blood pressure and haemodynamics in patients with morbid obesity. *J Hypetens.* 2013;31(11):2220-9; discussion 9.
119. Mallamaci F, Leonardis D, Pizzini P, et al. Procalcitonin and the inflammatory response to salt in essential hypertension: a randomized cross-over clinical trial. *J Hypetens.* 2013;31(7):1424-30.
120. Allen AR, Gullixson LR, Wolhart SC, et al. Dietary sodium influences the effect of mental stress on heart rate variability: A randomized trial in healthy adults. *J Hypetens.* 2014;32(2):374-82.
121. Cavka A, Cosic A, Jukic I, et al. The role of cyclo-oxygenase-1 in high-salt diet-induced microvascular dysfunction in humans. *J Physiol.* 2015;593(24):5313-24.
122. Gijsbers L, Dower JI, Schalkwijk CG, et al. Effects of sodium and potassium supplementation on endothelial function: A fully controlled dietary intervention study. *Br J Nutr.* 2015;114(9):1419-26.
123. He FJ, Wu Y, Feng XX, et al. School based education programme to reduce salt intake in children and their families (School-EduSalt): Cluster randomised controlled trial. *BMJ.* 2015;350 (no pagination)(h770).
124. Pinjuh MN, Rumboldt M, Rumboldt Z. Emphasized warning reduces salt intake: A randomized controlled trial. *J Am Soc Hypertens.* 2015;9(3):214-20.
125. Matthews EL, Brian MS, Ramick MG, et al. High dietary sodium reduces brachial artery flow-mediated dilation in humans with salt-sensitive and salt-resistant blood pressure. *J Appl Physiol.* 2015;: 1985). 118(12):1510-5.
126. Riphagen IJ, Gijsbers L, van Gastel MD, et al. Effects of potassium supplementation on markers of osmoregulation and volume regulation: results of a fully controlled dietary intervention study. *J Hypetens.* 2016;34(2):215-20.

127. Suckling RJ, He FJ, Markandu ND, et al. Modest salt reduction lowers blood pressure and albumin excretion in impaired glucose tolerance and type 2 diabetes mellitus: A randomized double-blind trial. *Hypertension.* 2016;67(6):1189-95.
128. Brian MS, Dalpiaz A, Matthews EL, et al. Dietary sodium and nocturnal blood pressure dipping in normotensive men and women. *J Hum Hypertens.* 2017;31(2):145-50.
129. Gefke M, Christensen NJ, Bech P, et al. Hemodynamic responses to mental stress during salt loading. *Clin Physiol Funct Imaging.* 2017;37(6):688-94.
130. Babcock MC, Brian MS, Watso JC, et al. Alterations in dietary sodium intake affect cardiovagal baroreflex sensitivity. *Am J Physiol Regul Integr Comp Physiol.* 2018;315(4):R688-R95.
131. Parvanova A, Trillini M, Podesta MA, et al. Moderate salt restriction with or without paricalcitol in type 2 diabetes and losartan-resistant macroalbuminuria (PROCEED): a randomised, double-blind, placebo-controlled, crossover trial. *Lancet Diabetes Endocrinol.* 2018;6(1):27-40.
132. Rorije NMG, Olde Engberink RHG, Chahid Y, et al. Microvascular Permeability after an Acute and Chronic Salt Load in Healthy Subjects: A Randomized Open-label Crossover Intervention Study. *Anesthesiology.* 2018;128(2):352-60.
133. Wang Y, Chu C, Wang KK, et al. Effect of Salt Intake on Plasma and Urinary Uric Acid Levels in Chinese Adults: An Interventional Trial. *Sci Rep.* 2018;8(1):1434.
